# Supplementary material for: Understanding the AIE phenomenon of nonconjugated rhodamine derivatives via aggregation-induced molecular conformation change
Source: Nat Commun. 2024 Feb 2;15:999. doi: 10.1038/s41467-024-45271-6 (PMC10837119; doi:10.1038/s41467-024-45271-6)
Supplement: Supplementary file 1 — Supplementary Information [file 41467_2024_45271_MOESM1_ESM.pdf]

Supplementary information

Understanding the AIE phenomenon of nonconjugated rhodamine derivatives via aggregation-induced molecular conformation change

Lin-Lin Yang[1,2,5], Haoran Wang[2,3,4,5], Jianyu Zhang[3], Bo Wu[2], Qiyao Li[2], Jie-Ying Chen[1], A-Ling Tang[1], Jacky W. Y. Lam[3], Zheng Zhao[2,4,*], Song Yang[1,*], and Ben Zhong Tang[2,3,4,*]

[1]National Key Laboratory of Green Pesticide, Key Laboratory of Green Pesticide and Agricultural Bioengineering, Ministry of Education, Guizhou University, Huaxi District, Guiyang 550025, China.

[2]Clinical Translational Research Center of Aggregation-Induced Emission, The Second Affiliated Hospital, School of Science and Engineering, Shenzhen Institute of Aggregate Science and Technology, The Chinese University of Hong Kong, Shenzhen (CUHK-Shenzhen), Guangdong 518172, P.R. China.

[3]Hong Kong Branch of Chinese National Engineering Research Center for Tissue Restoration and Reconstruction, Department of Chemistry, The Hong Kong University of Science and Technology, Clear Water Bay, Kowloon, Hong Kong, 999077, China.

[4]HKUST Shenzhen Research Institute, No. 9 Yuexing 1st RD, South Area Hi-tech Park, Nanshan, Shenzhen, 518057, China.

[5]These authors contributed equally: Lin-Lin Yang, Haoran Wang.

*Correspondence authors: zhaozheng@cuhk.edu.cn, jhzx.msm@gmail.com and tangbenz@cuhk.edu.cn.

Contents

1. Synthesis of BISX, ISX, MISX and MTSX 3

2. The DFT-calculated possible mechanism of MISX 6

3. The ^{1}H NMR, ^{13}C NMR and HRMS spectra of BISX, ISX, MISX and MTSX 7

4. Crystallographic data and structures of BISX, ISX, MISX and MTSX 13

5. Photophysical properties and DLS of BISX, ISX, MISX and MTSX 14

6. The PL spectra of BISX, ISX, MISX and MTSX in methanol-glycerol mixture with different glycerol fractions. 17

7. PL spectra of BISX, ISX, MISX and MTSX in THF at different temperature 17

8. Synthesis of ASX, BIPM, IPM, MIPM and MTPM 18

9. The ^{1}H NMR, ^{13}C NMR and HRMS spectra of ASX, BIPM, IPM, MIPM and MTPM 21

10. PL spectra of ASX, BIPM, IPM, MIPM and MTPM 28

11. Excitation spectra of BISX, ISX, MISX and MTSX 29

12. Intermolecular interactions of BISX, ISX, MISX and MTSX crystals 30

13. The UV-vis absorbance spectra in the film of BISX, ISX, MISX and MTSX in PMMA31

14. Theoretical calculation 32

15. BISX treated with TFA and TEA 33

16. Application in the detection of amines released by clams, crayfish and fish spoilage 33

1. Synthesis of BISX, ISX, MISX and MTSX

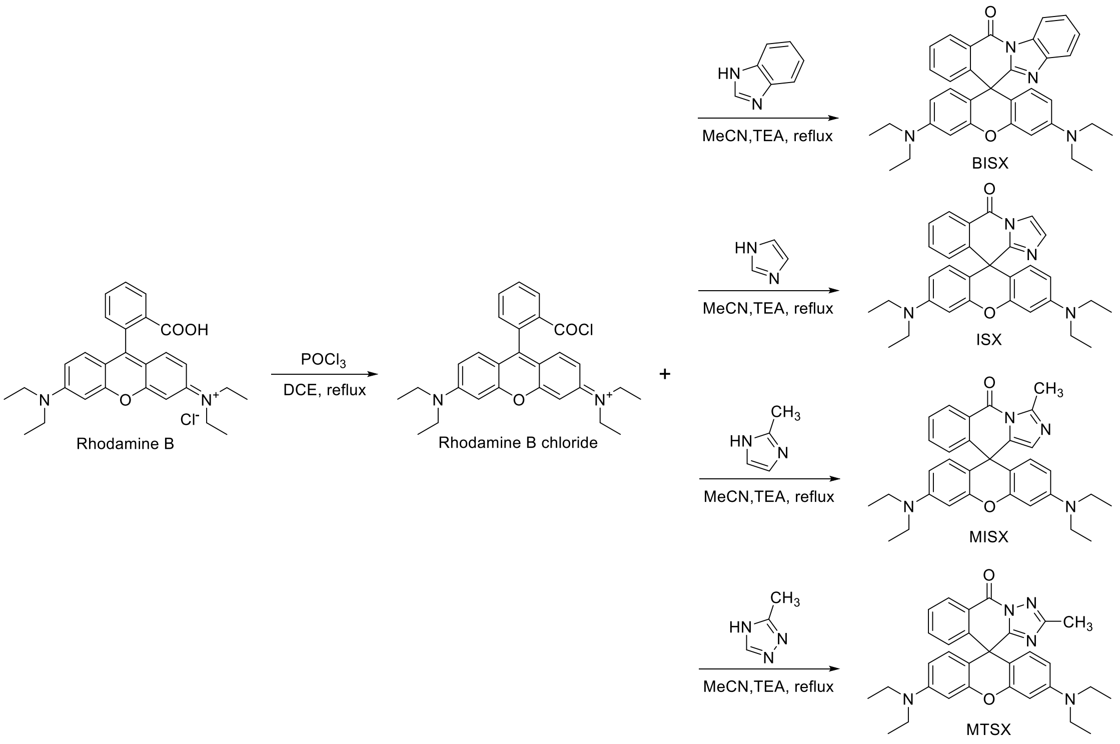

Supplementary Fig. 1 Synthetic route to BISX, ISX, MISX and MTSX.

Synthesis of 3',6'-bis(diethylamino)-11H-spiro[benzo[4,5]imidazo[1,2-b]isoquinoline-6,9'-xanthen]-11-one (BISX)

Rhodamine B (200 mg, 0.4 mmol) was dissolved in 10 mL anhydrous 1,2-dichloroethane and 0.4 mL phosphorus oxychloride. The mixture was refluxed for 6 h and concentrated by evaporation under reduced pressure. The obtained crude rhodamine acid chloride was dissolved in 10 mL anhydrous acetonitrile, slowly adding dropwise to the mixture of benzimidazole (71 mg, 0.6 mmol) and anhydrous acetonitrile (5 mL) containing 0.5 mL anhydrous TEA, and then refluxed for 4 h. The solvent was removed under reduced pressure to give a red oil, and the crude residue was purified by silica gel column chromatography (petroleum ether–ethyl acetate, 5/1~2/1) to give 95 mg light beige solid, yield 43.7 %. The crystal was grown from its acetonitrile solution and detected by Bruker Smart Apex II CCD diffractometer. ^{1}H NMR (400 MHz, $CDCl_3$) δ 8.53 (d, J = 7.7 Hz, 1H), 8.43 (dd, J = 7.9, 1.3 Hz, 1H), 7.71 (d, J = 7.7 Hz, 1H), 7.57 – 7.49 (m, 1H), 7.47 – 7.41

(m, 1H), 7.39 (dd, *J* = 7.9, 1.1 Hz, 1H), 7.34 (td, *J* = 7.7, 1.3 Hz, 1H), 7.29 (d, *J* = 7.9 Hz, 1H), 6.47 (d, *J* = 2.5 Hz, 2H), 6.36 (d, *J* = 8.8 Hz, 2H), 6.18 (dd, *J* = 8.8, 2.6 Hz, 2H), 3.30 (q, *J* = 7.1 Hz, 8H), 1.13 (t, *J* = 7.0 Hz, 12H). ^{13}C NMR (100 MHz, $CDCl_3$） δ 159.8, 158.8, 150.1, 147.8, 147.1, 142.3, 133.7, 130.1, 129.9, 128.4, 126.5, 126.1, 124.0, 123.6, 123.3, 119.1, 114.6, 106.7, 97.5, 43.9, 43.2, 11.5. HRMS (ESI) m/z: $[M + H]^+$ Calcd for $C_{35}H_{35}N_4O_2$ 543.2754; Found 543.2744.

Synthesis of 3',6'-bis(diethylamino)-5H-spiro[imidazo[1,2-b]isoquinoline-10,9'-xanthen]-5-one (ISX)

Rhodamine B (200 mg, 0.4 mmol) was dissolved in 10 mL anhydrous 1,2-dichloroethane and 0.4 mL phosphorus oxychloride. The obtained mixture was refluxed for 6 h and concentrated by evaporation under the reduced pressure. The obtained crude rhodamine acid chloride was dissolved in 10 mL anhydrous acetonitrile, slowly adding dropwise to the mixture of imidazole (41 mg, 0.6 mmol) and anhydrous acetonitrile (5 mL) containing 0.5 mL anhydrous TEA, and then refluxed for 4 h. The solvent was removed under reduced pressure to give a red oil, and the crude residue was purified by silica gel column chromatography (petroleum ether–ethyl acetate, 5/1~1/1) to give 103 mg light-yellow solid, yield 52.2 %. The crystal was grown from its acetonitrile solution and detected by Bruker Smart Apex II CCD diffractometer. ^{1}H NMR (400 MHz, $CDCl_3$) δ 8.37 (dd, *J* = 7.9, 1.2 Hz, 1H), 7.70 (d, *J* = 1.6 Hz, 1H), 7.60 – 7.48 (m, 1H), 7.45 – 7.39 (m, 1H), 7.31 (d, *J* = 8.0 Hz, 1H), 7.18 – 7.12 (m, 1H), 6.45 (d, *J* = 2.5 Hz, 2H), 6.27 (d, *J* = 8.8 Hz, 2H), 6.18 (d, *J* = 2.5 Hz, 1H), 3.30 (q, *J* = 7.1 Hz, 8H), 1.13 (t, *J* = 7.1 Hz, 12H). ^{13} C NMR (100 MHz, $CDCl_3$) δ 159.5, 155.3, 151.1, 149.5, 147.8, 134.8, 131.2, 131.1, 128.9, 127.7, 126.9, 123.7, 112.4, 111.1, 107.5, 98.3, 44.2, 44.0, 12.3. HRMS (ESI) m/z: $[M + H]^+$ Calcd for $C_{31}H_{33}N_4O_2$ 493.2598; Found 493.2588.

Synthesis of 3',6'-bis(diethylamino)-3-methyl-5H-spiro[imidazo[1,5-b]isoquinoline-10,9'-xanthen]-5-one (MISX)

MISX was prepared according to literature method (*Org. Lett.* **2020**, *22*, 8234-8239).

Rhodamine B (288 mg, 0.6 mmol) was dissolved in 15 mL anhydrous 1,2-dichloroethane, then 0.5 mL phosphorus oxychloride was added into the above-mentioned solution. The obtained mixture was refluxed for 8 h and concentrated by evaporation under the reduced pressure. The obtained crude rhodamine acid chloride was dissolved in 10 mL anhydrous acetonitrile, slowly adding dropwise to the mixture of 2-methylimidazole (123 mg, 1.5 mmol) and anhydrous acetonitrile (5 mL) containing 0.5 mL anhydrous TEA, and then refluxed for 6 h. The solvent was removed under reduced pressure to give a red oil, and the crude product was purified by silica gel column chromatography (petroleum ether–ethyl acetate, 100/1~60/1) to give 160.0 mg light green solid, yield 52.5%. The crystal was grown from its acetonitrile-water (4:6, V/V) solution and detected by Bruker Smart Apex II CCD diffractometer. ^{1}H NMR (500 MHz, $CDCl_3$) δ 8.34 (dd, J = 8.0, 1.3 Hz, 1H), 7.50 – 7.45 (m, 1H), 7.39 – 7.34 (m, 1H), 7.18 (dd, J = 8.0, 0.7 Hz, 1H), 6.47 (d, J = 3.0 Hz, 2H), 6.46 (s, 1H), 6.39 (d, J = 2.6 Hz, 2H), 6.22 (dd, J = 8.8, 2.7 Hz, 2H), 3.31 (q, J = 7.1 Hz, 8H), 2.87 (s, 3H), 1.15 (t, J = 7.1 Hz, 12H). ^{13}C NMR (125 MHz) δ 160.8, 150.8, 149.8, 147.8, 145.9, 139.2, 135.3, 136.6, 130.0, 128.5, 127.3, 126.2, 124.8, 113.3, 108.2, 98.0, 44.6, 41.3, 17.6, 12.6. HRMS (ESI) m/z: $[M + H]^+$ Calcd for $C_{32}H_{35}N_4O_2$ 507.2755; Found 508.2749.

Synthesis of 3,6-bis(diethylamino)-2'-methyl-5'H-spiro[xanthene-9,10'-[1,2,4]triazolo[1,5-b]isoquinolin]-5'-one (MTSX)

Rhodamine B (200 mg, 0.4 mmol) was dissolved in 10 mL anhydrous 1,2-dichloroethane and 0.4 mL phosphorus oxychloride. The obtained mixture was refluxed for 6 h and concentrated by evaporation under reduced pressure. The obtained crude rhodamine acid chloride was dissolved in10 mL anhydrous acetonitrile, slowly dropwise to the mixture 3-methyl-1H-1,2,4-triazole (50 mg, 0.6 mmol) and anhydrous acetonitrile (5 mL) containing 0.5 mL anhydrous TEA, and then refluxed for 4 h. The solvent was removed under reduced pressure to give a red oil, and the crude residue was purified by silica gel column chromatography (petroleum ether–ethyl acetate, 10/1~2/1) to give 111 mg light green solid, yield 54.7 %. The crystal was grown from its acetonitrile solution and detected by Bruker Smart Apex II CCD diffractometer. ^{1}H NMR (400 MHz, $CDCl_3$) δ 8.42 (dd, J = 7.9, 1.3 Hz,

1H), 7.57 – 7.50 (m, 1H), 7.47 – 7.40 (m, 1H), 7.28 (d, *J* = 0.7 Hz, 1H), 6.44 (d, *J* = 2.5 Hz, 2H), 6.26 (d, *J* = 8.8 Hz, 2H), 6.19 (dd, *J* = 8.8, 2.6 Hz, 2H), 3.31 (q, *J* = 7.1 Hz, 8H), 2.40 (s, 3H), 1.14 (t, *J* = 7.1 Hz, 12H). ^{13}CNMR (100 MHz, $CDCl_3$) δ 163.2, 162.4, 155.2, 149.1, 146.9, 146.3, 133.1, 129.1, 127.1, 126.2, 125.4, 121.4, 124.9, 107.4, 105.7, 96.5, 42.5, 42.1, 12.3, 10.4. HRMS (ESI) m/z: $[M + H]^+$ Calcd for $C_{31}H_{34}N_5O_2$ 508.2707; Found 508.2695.

2. The DFT-calculated possible mechanism of MISX

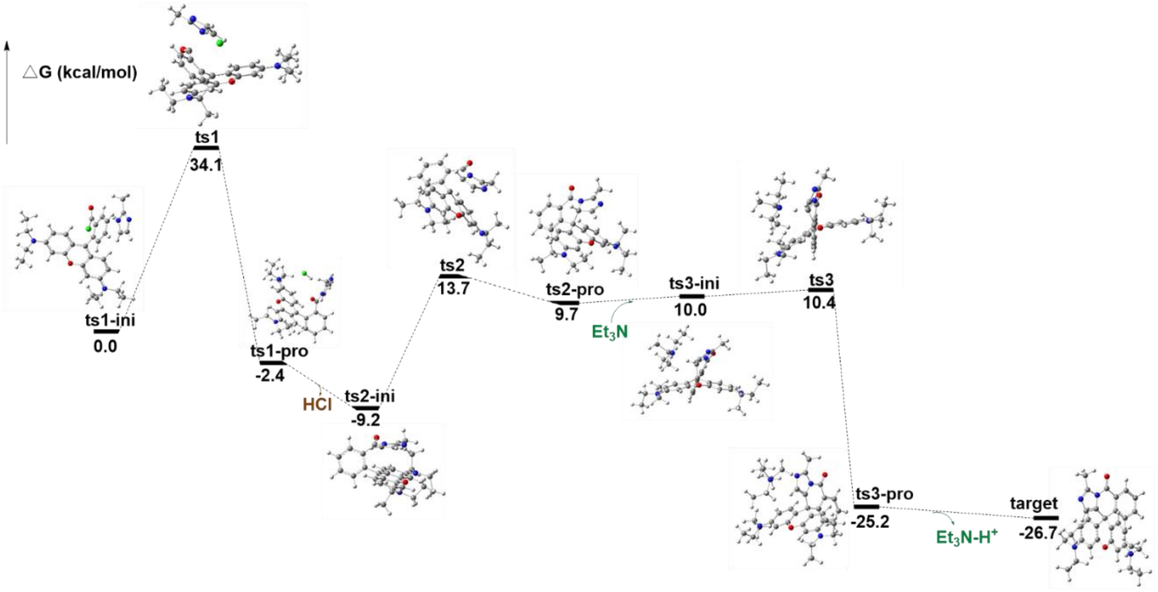

Supplementary Fig. 2 Gibbs free energy profile for the DFT-calculated possible mechanism of MISX.

3. The ^{1}H NMR, ^{13}C NMR and HRMS spectra of BISX, ISX, MISX and MTSX

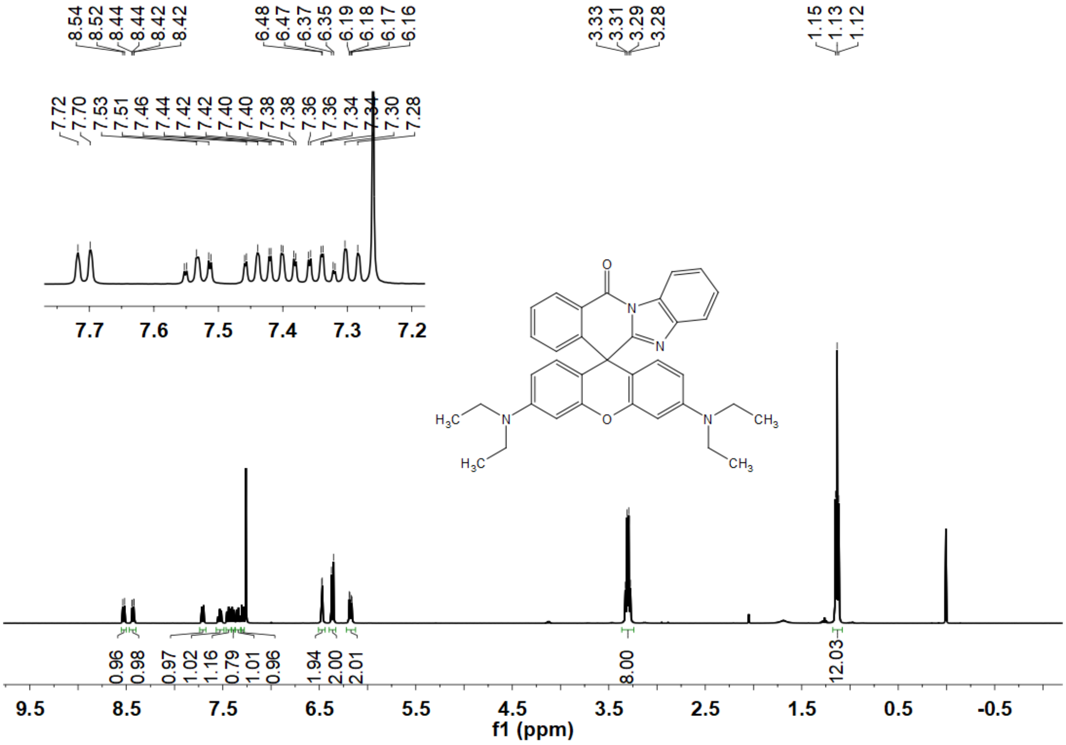

Supplementary Fig. 3 ^{1}H NMR of BISX in $CDCl_3$ (400 MHz).

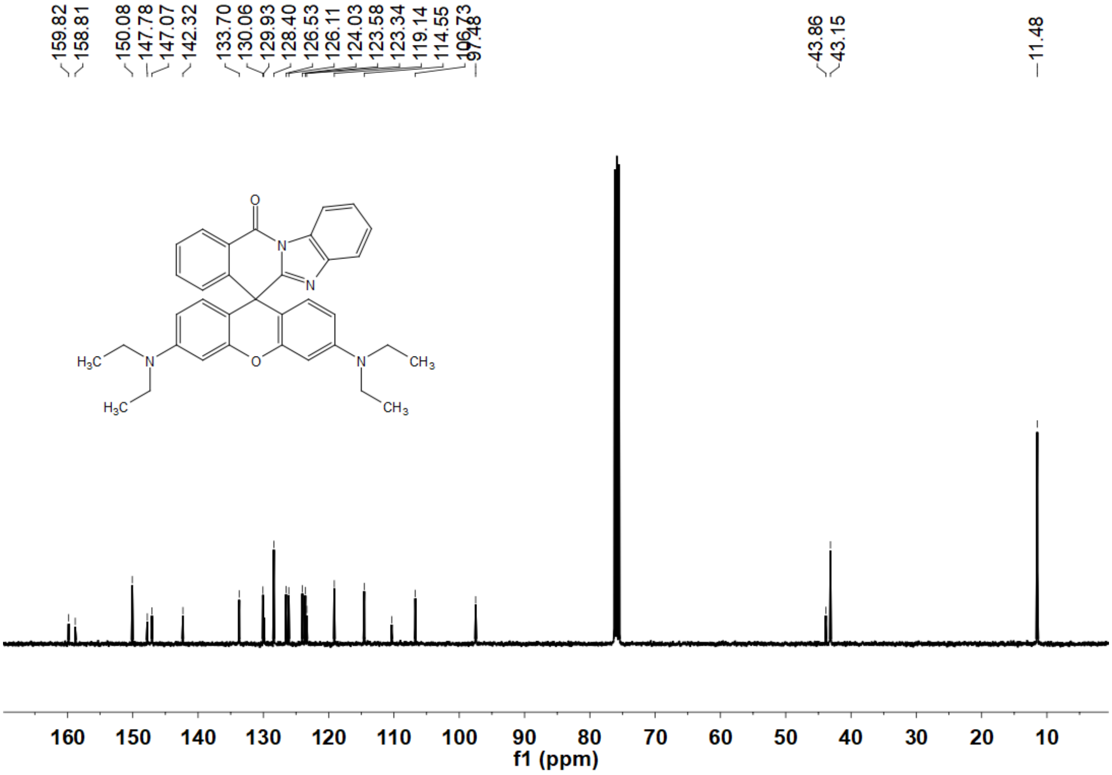

Supplementary Fig. 4 ^{13}C NMR of BISX in $CDCl_3$ (100 MHz).

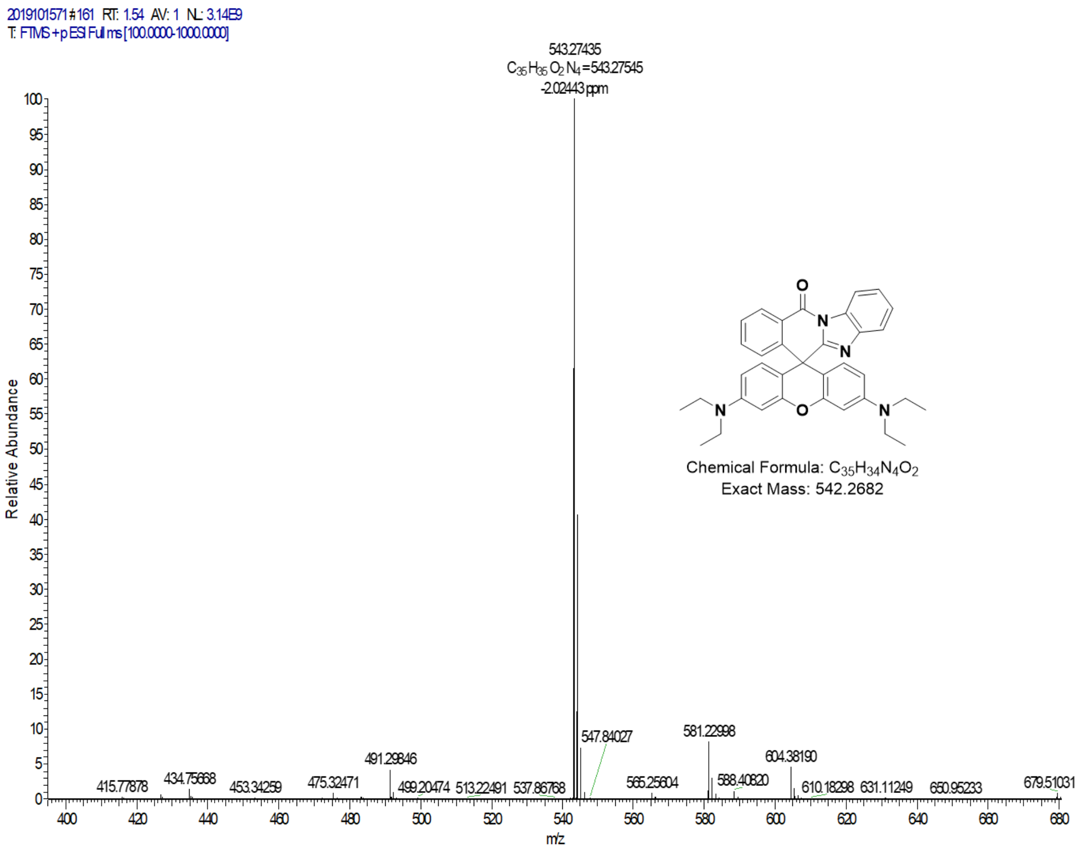

Supplementary Fig. 5 HRMS spectrum of BISX.

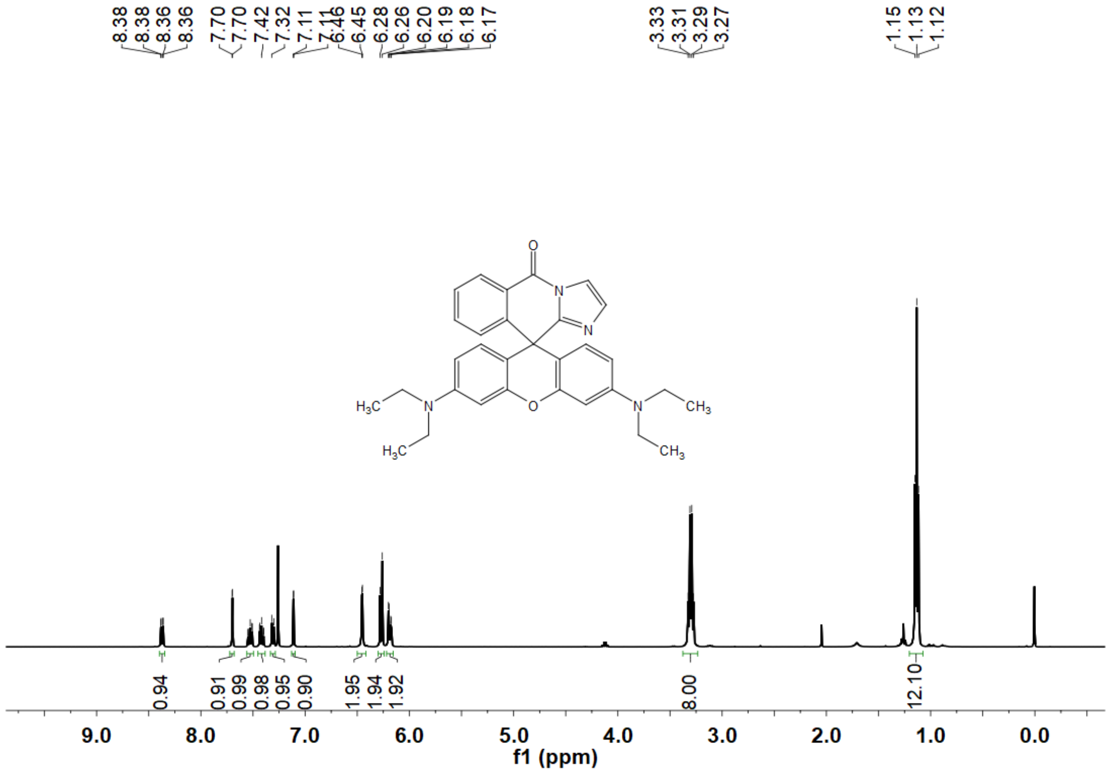

Supplementary Fig. 6 ^{1}H NMR of ISX in $CDCl_3$ (400 MHz).

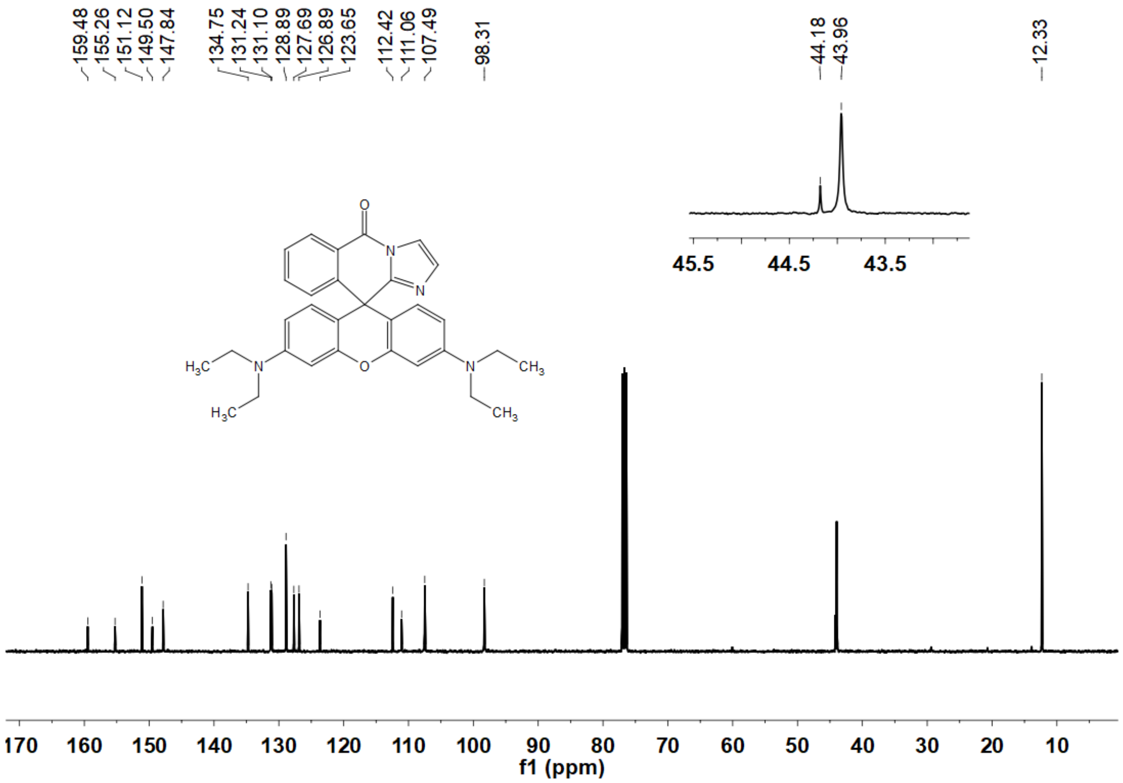

Supplementary Fig. 7 ^{13}C NMR of ISX in $CDCl_3$ (100 MHz).

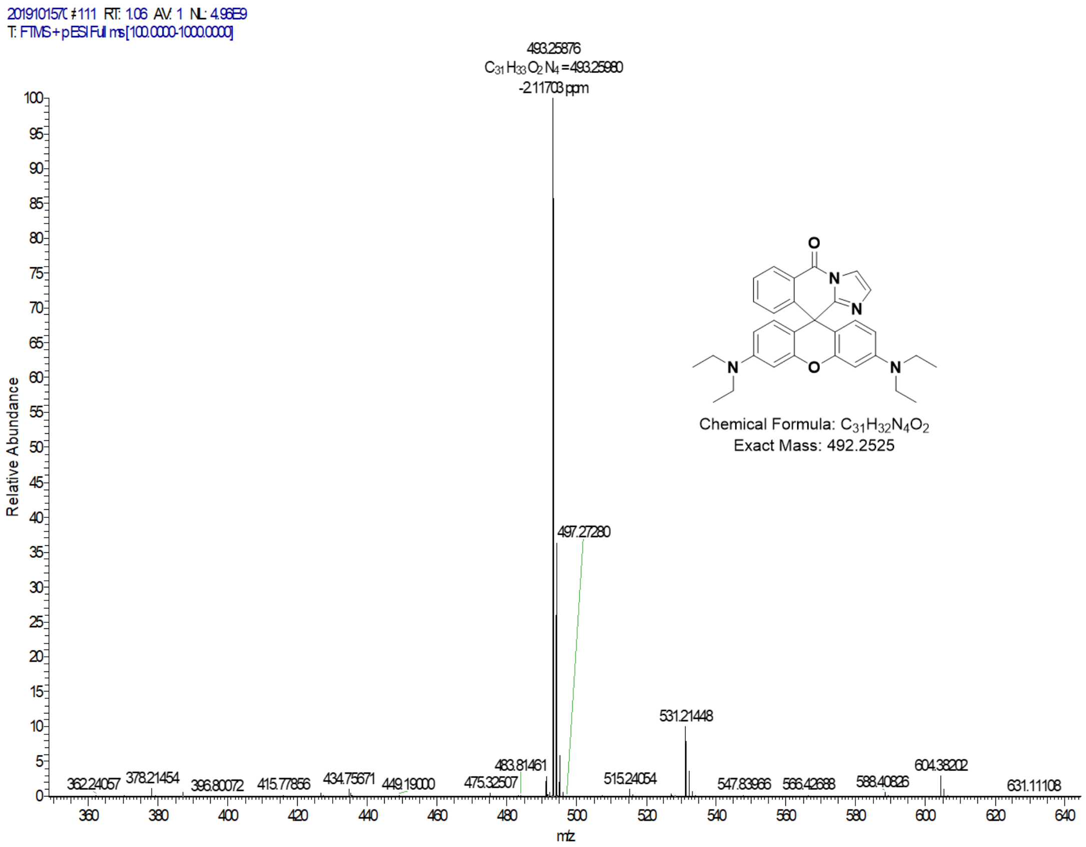

Supplementary Fig. 8 HRMS spectrum of ISX.

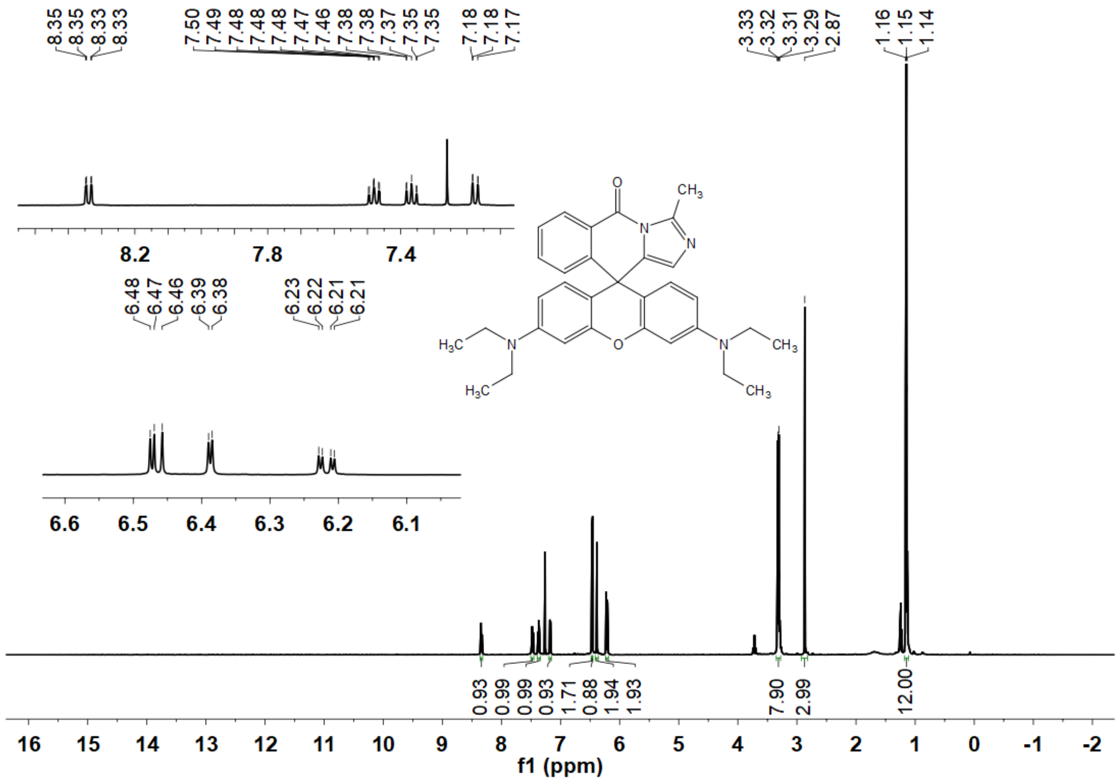

Supplementary Fig. 9 ^{1}H NMR of MISX in $CDCl_3$ (500 MHz).

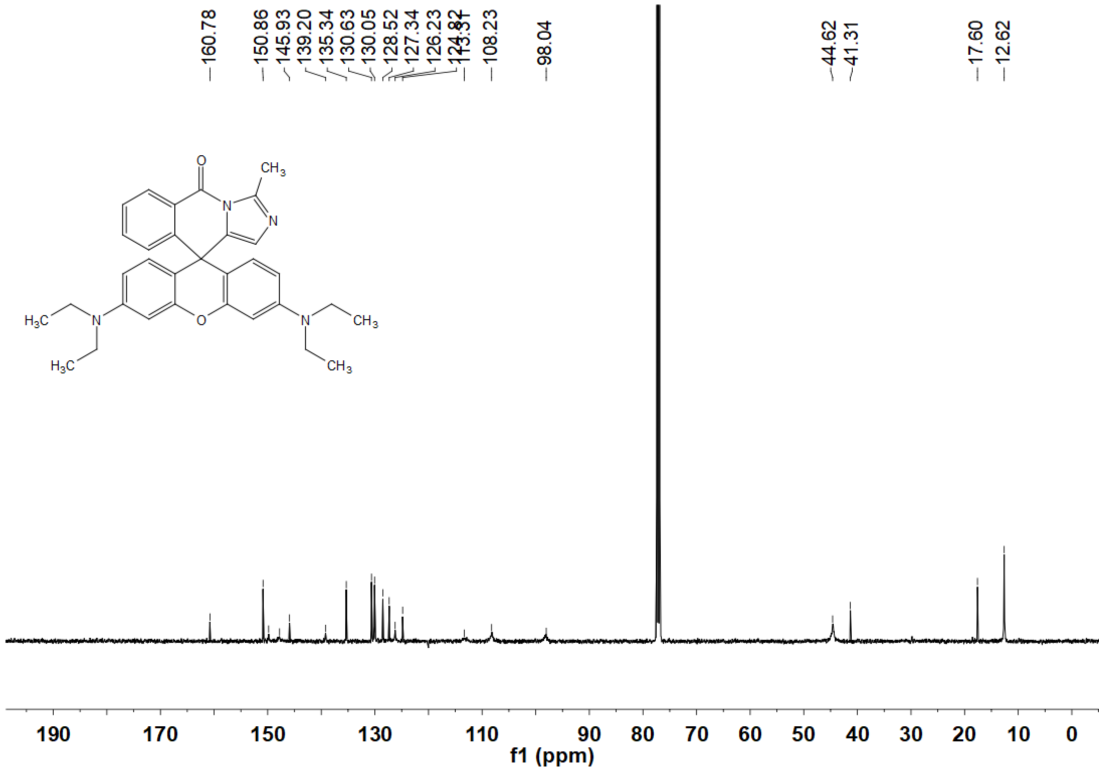

Supplementary Fig. 10 ^{13}C NMR of MISX in $CDCl_3$ (125 MHz).

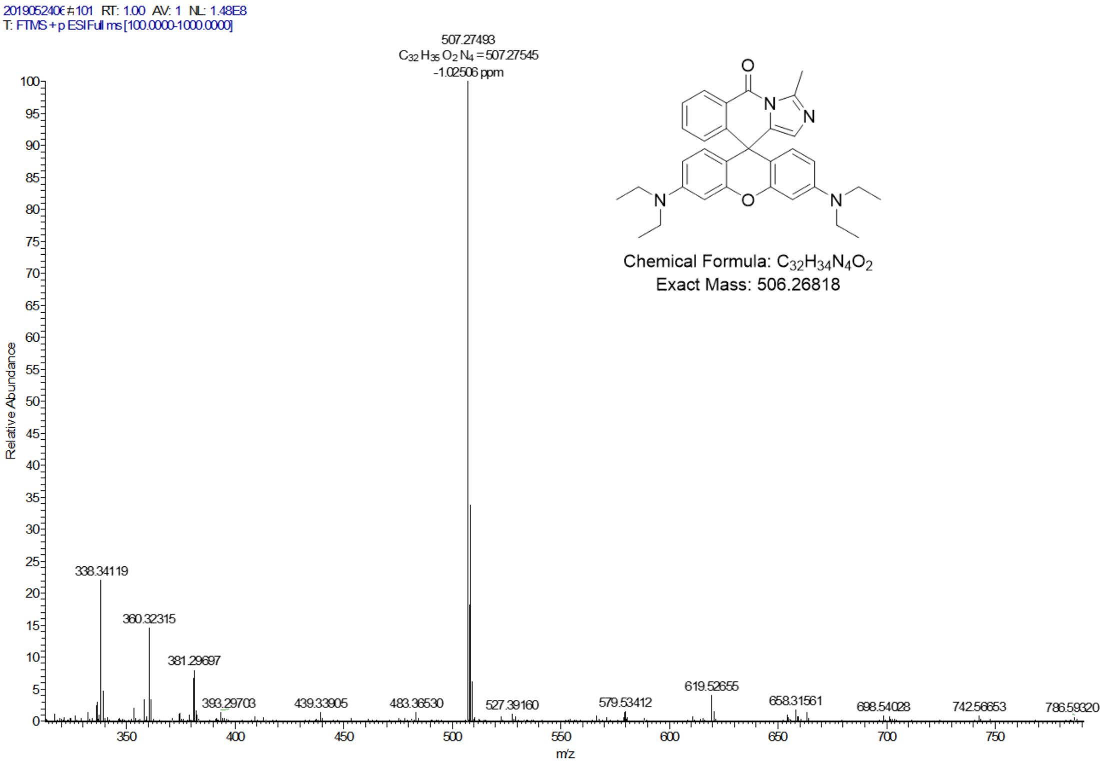

Supplementary Fig. 11 HRMS spectrum of MISX.

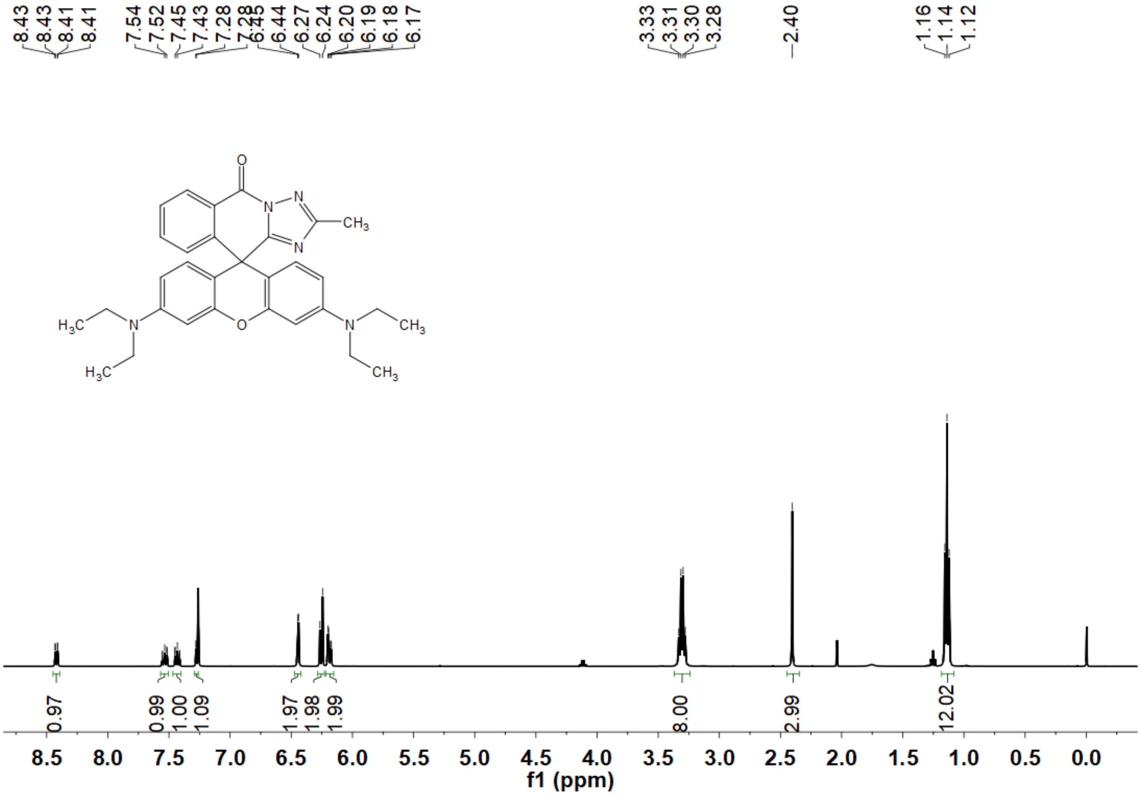

Supplementary Fig. 12 ^{1}H NMR of MTSX in $CDCl_3$ (400 MHz).

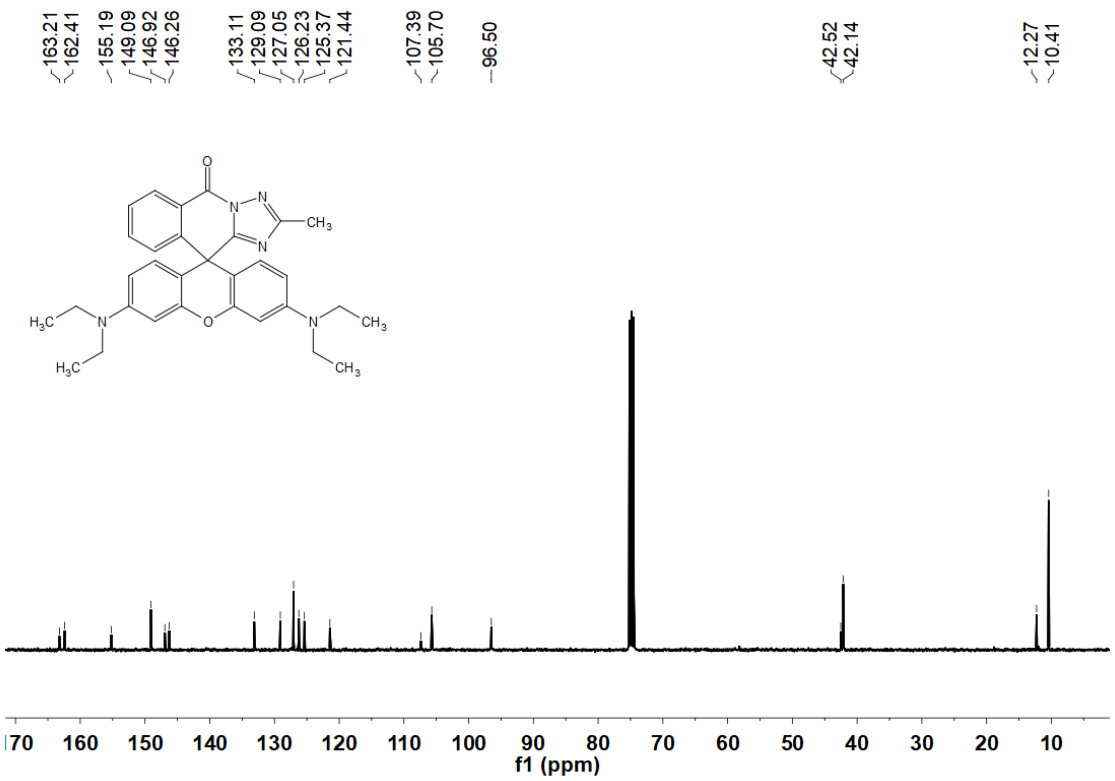

Supplementary Fig. 13 ^{13}C NMR of MTSX in $CDCl_3$ (100 MHz).

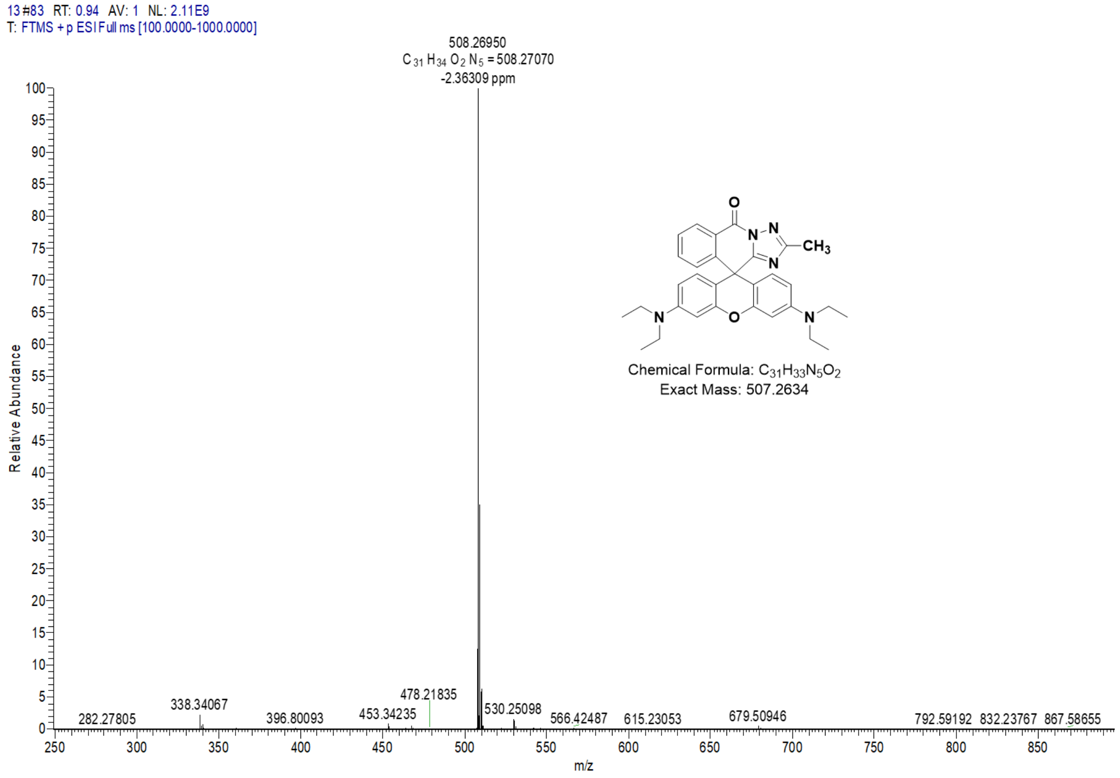

Supplementary Fig. 14 HRMS spectrum of MTSX.

4. Crystallographic data and structures of BISX, ISX, MISX and MTSX

Supplementary Tab.1 Crystal data and structure refinement parameters of BISX, ISX, MISX and MTSX.

Name	BISX	ISX	MISX	MTSX
Formula	$C_{35}H_{34}N_4O_2$	$C_{31}H_{32}N_4O_2$	$C_{33}H_{34}N_4O_2$	$C_{31}H_{33}N_5O_2$
CCDC numbers	2051533	2051534	1991171	2051536
Crystal system	Monoclinic	Monoclinic	Monoclinic	Monoclinic
Space group	P2(1)/c	C2/c	P2(1)/c	P2(1)/c
a (Å)	15.9652(14)	37.443(3)	10.9840(9)	23.291(2)
b (Å)	11.9611(11)	12.1792(11)	13.9551(12)	8.8408(6)
c (Å)	15.2259(14)	23.007(2)	35.580(3)	29.234(3)
α (°)	90	90	90.00	90
β (°)	95.467(2)	100.166(2)	99.240(3)	107.791(4)
γ (°)	90	90	90.00	90
V (Å^3)	2894.3(5)	10327.3	5383.1(8)	5731.9(8)
Z	4	16	8	8
Calculated density (g·cm^{-3})	1.245	1.267	1.249	1.269
F (000)	1152	4192	2156	2328
Reflections	13908 / 5093	25726 / 9102	9397 / 9397	28100 / 10101
collected/unique	[R(int) = 0.0490]	[R(int) = 0.0866]	[R(int) = 0.0000]	[R(int) = 0.0829]
Goodness-of-fit on F^2	1.059	1.052	1.078	1.059
Final *R* indices	R_1 = 0.0510	R_1 = 0.0700	R_1 = 0.1258	R_1 = 0.0814
[I >2$\sigma(I)$]	wR_2 = 0.1012	wR_2 = 0.1118	wR_2 = 0.2719	wR_2 = 0.1857

R indices (all data)	R_1 = 0.0856, wR_2 = 0.1092	R_1 = 0.2208, wR_2 = 0.1337	R_1 = 0.3143, wR_2 = 0.3226	R_1 = 0.1822, wR_2 = 0.2173

5. Photophysical properties and DLS of BISX, ISX, MISX and MTSX

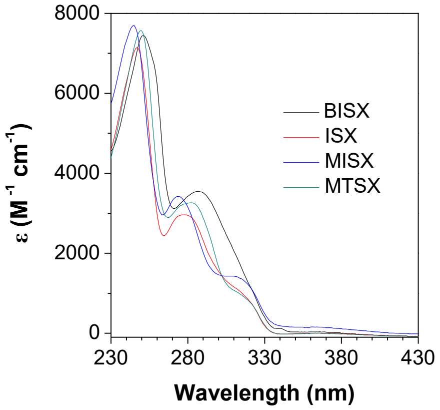

Supplementary Fig. 15 UV-vis absorbance spectrum of BISX, ISX, MISX, and MTSX in THF. Concentration (c) = 1×10^{-5} M.

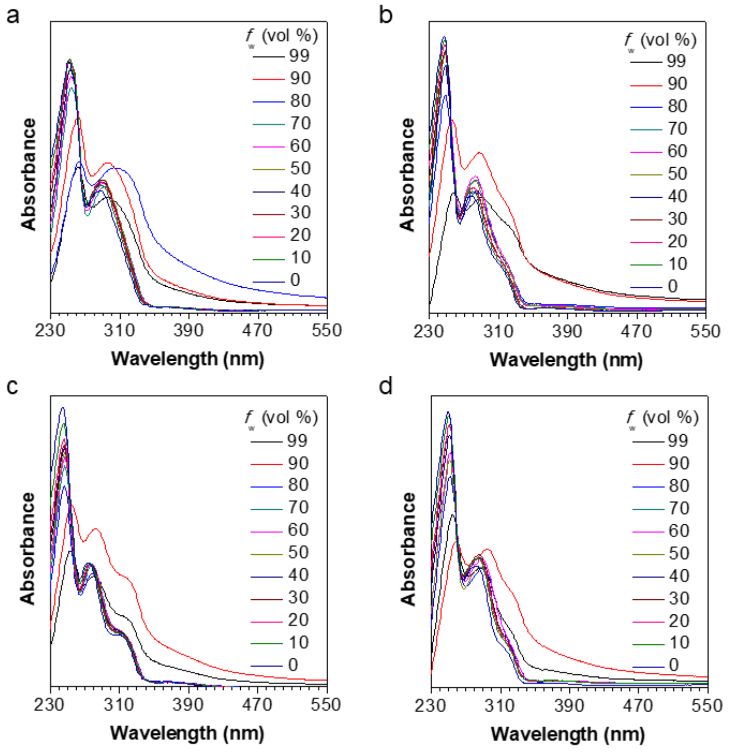

Supplementary Fig. 16 (a-d) UV-vis absorbance spectra of BISX, ISX, MISX and MTSX in THF/water mixtures with different water fractions (f_w). Concentration (c) = 1×10^{-5} M, excitation wavelength (λ_{ex}) = 365 nm.

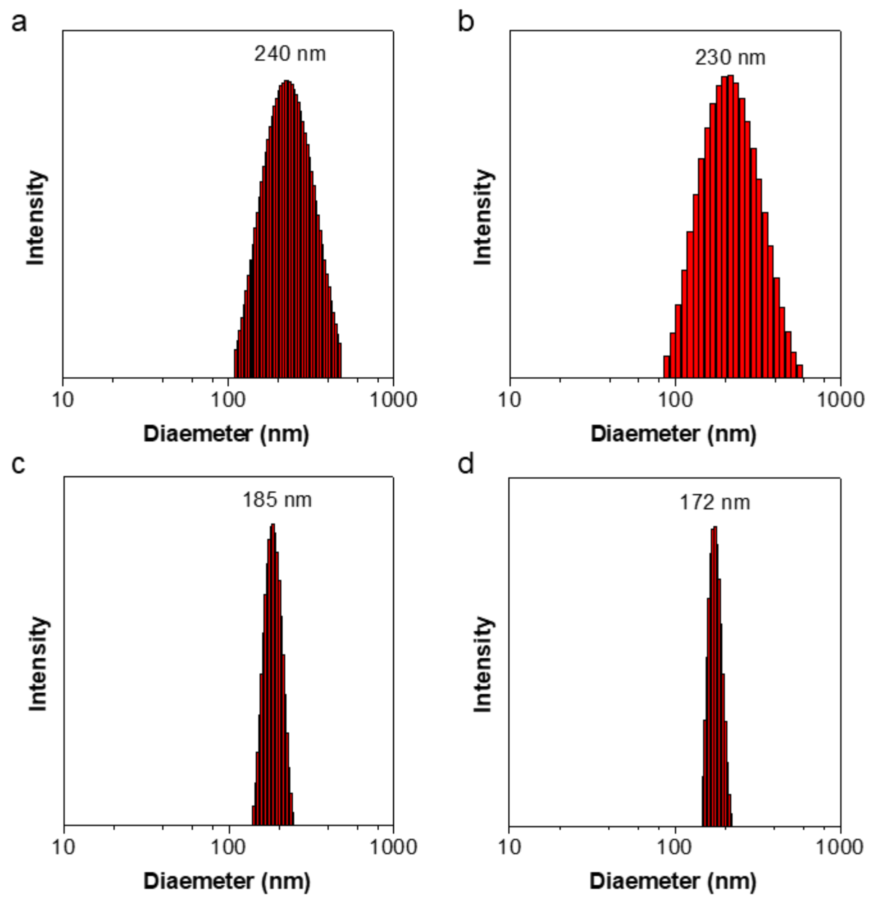

Supplementary Fig. 17 (a-d) DLS of BISX, ISX, MISX and MTSX in THF/water mixtures with water fraction = 99%. Concentration (c) = 1×10^{-5} M.

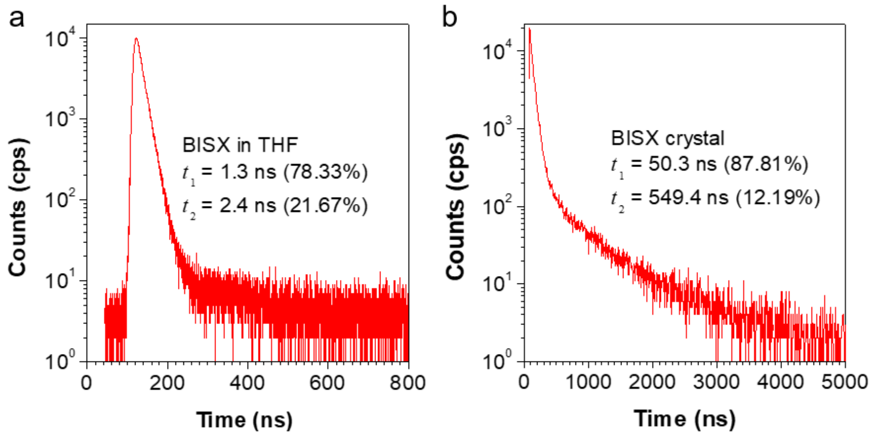

Supplementary Fig. 18 (a) Time-resolved fluorescence decay curves of BISX in THF at 550 nm, Concentration (c) = 1×10^{-5} M. (b) Time-resolved fluorescence decay curves of BISX crystal at 475 nm.

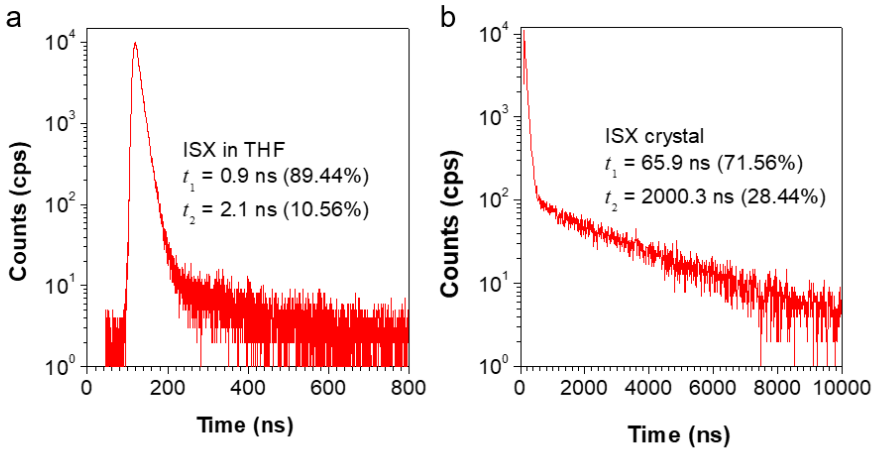

Supplementary Fig. 19 (a) Time-resolved fluorescence decay curves of ISX in THF at 550 nm, Concentration (*c*) = 1×10^{-5} M. (b) Time-resolved fluorescence decay curves of ISX crystal at 515 nm.

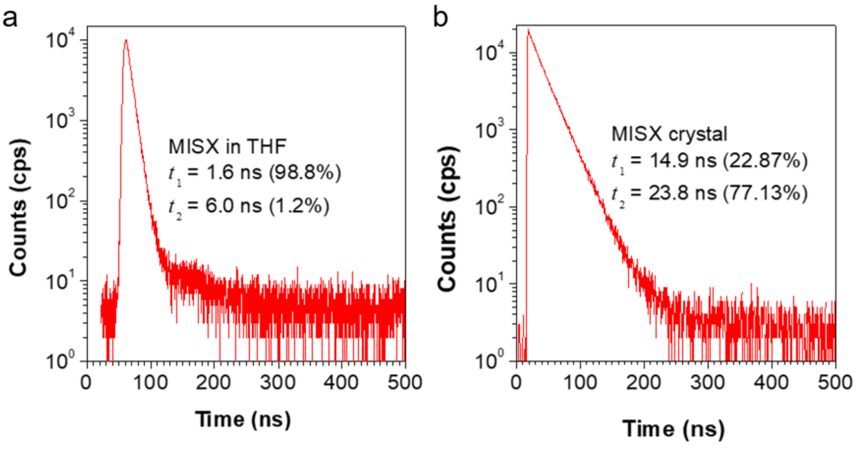

Supplementary Fig. 20 (a) Time-resolved fluorescence decay curves of MISX in THF at 550 nm, Concentration (*c*) = 1×10^{-5} M. (b) Time-resolved fluorescence decay curves of MISX crystal at 478 nm.

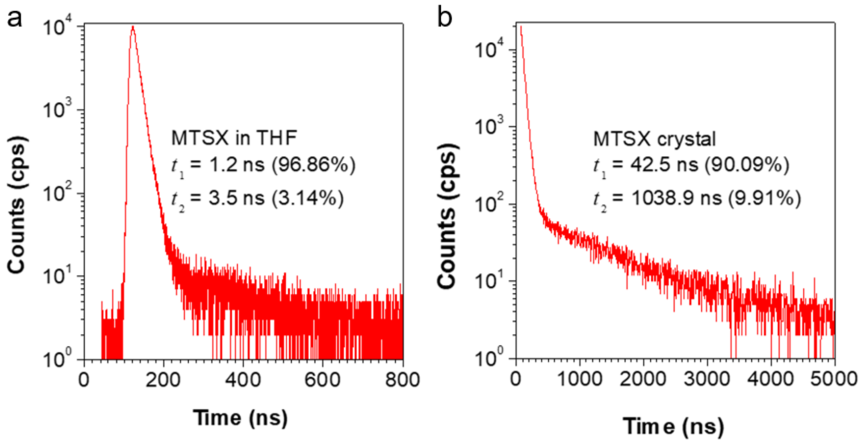

Supplementary Fig. 21 (a) Time-resolved fluorescence decay curves of MTSX in THF at 550 nm, Concentration (*c*) = 1×10^{-5} M. (b) Time-resolved fluorescence decay curves of MTSX crystal at 497 nm.

Supplementary Tab. 2 Photophysical properties of BISX, ISX, MISX and MTSX.

Name	λ_{abs}/ nm	λ_{em}/nm		Φ_F (%)		
		Agg[a]	Cry[c]	Agg[a]	Soln[b]	Cry[c]
BISX	365	505	475	23.2	< 0.1	64.1
ISX	365	518	515	19.6	< 0.1	46.0
MISX	365	504	478	17.5	< 0.1	69.4
MTSX	365	516	497	11.9	< 0.1	72.7

Emission wavelength (λ_{em}) : PL maximum. Agg[a] : in THF/water mixtures with water fractions = 99%, Concentration (c) = 1×10^{-5} M. Soln[b] = in THF, c = 1×10^{-5} M. Cry[c] : crystal. Φ_F = absolute fluorescence quantum yield measured by an integrating sphere.

6. The PL spectra of BISX, ISX, MISX and MTSX in methanol-glycerol mixture with different glycerol fractions.

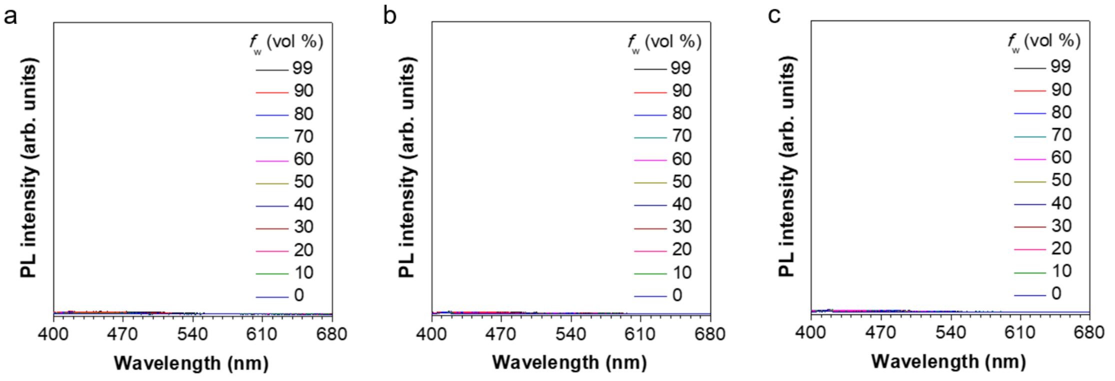

Supplementary Fig. 22 (a-c) PL spectra of ISX, MISX and MTSX in methanol/glycerol mixtures with different glycerol fractions (f_g). Concentration (c) = 1×10^{-5} M, excitation wavelength (λ_{ex}) = 365 nm.

7. PL spectra of BISX, ISX, MISX and MTSX in THF at different temperature

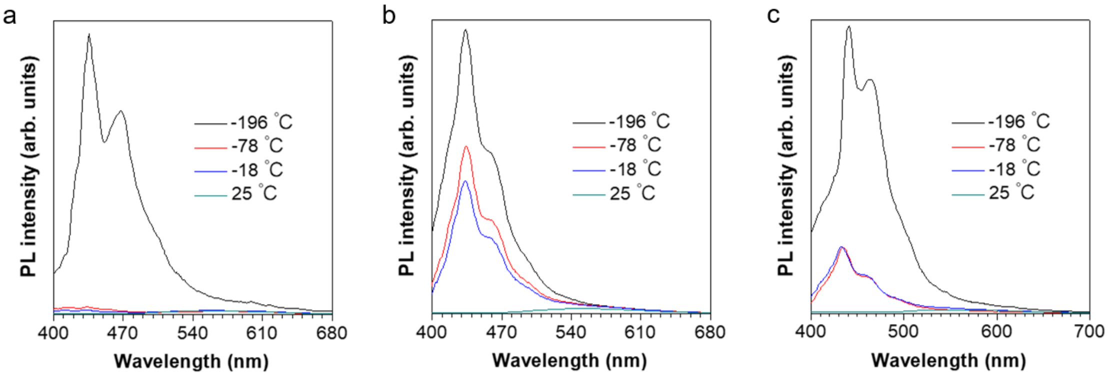

Supplementary Fig. 23 (a-c) PL spectra of ISX, MISX and MTSX in THF with different temperature. Concentration (c) = 1×10^{-5} M, excitation wavelength (λ_{ex}) = 365 nm.

8. Synthesis of ASX, BIPM, IPM, MIPM and MTPM

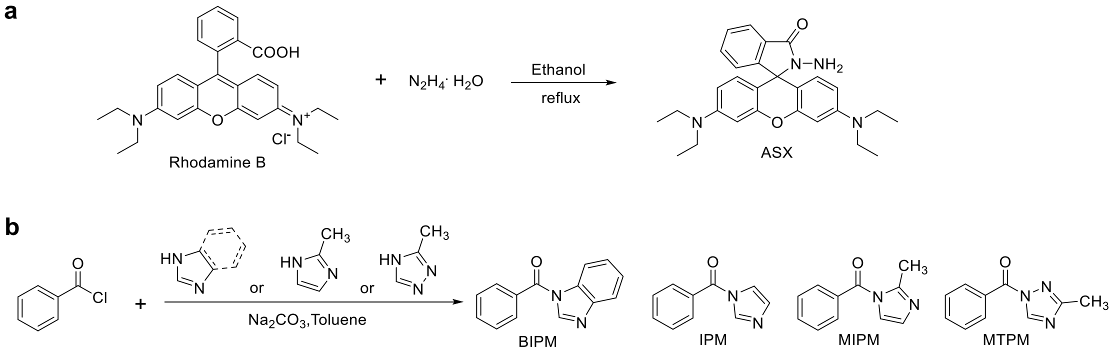

Supplementary Fig. 24 (a) Synthetic route to ASX. (b) Synthetic route to BIPM, IPM, MIPM and MTPM.

Synthesis of 2-amino-3',6'-bis(diethylamino)spiro[isoindoline-1,9'-xanthen]-3-one (ASX)

Rhodamine B (200 mg, 0.4 mmol) was dissolved in 10 mL ethanol and 0.1 mL (98%) hydrazine hydrate. The obtained mixture was refluxed for 8 h and concentrated by evaporation under reduced pressure, and the crude residue was purified by silica gel column chromatography (petroleum ether–ethyl acetate, 3/1~1/1) to give a light gray solid, 175 mg, yield 85.2 %. ^{1}H NMR (400 MHz, DMSO-d_6) δ 7.79 – 7.74 (m, 1H), 7.53 – 7.40 (m, 2H), 6.98 (dd, J = 5.2, 2.8 Hz, 1H), 6.35 (d, J = 17.2 Hz, 6H), 4.28 (s, 2H), 3.31 (dd, J = 14.0, 7.0 Hz, 8H), 1.08 (t, J = 6.9 Hz, 12H). ^{13}C NMR (100 MHz, DMSO-d_6) δ 165.8, 153.5, 152.4, 148.6, 132.9, 130.1, 128.6, 128.2, 124.0, 122.6, 108.2, 105.9, 97.9, 65.2, 44.2, 12.9. HRMS (ESI) m/z: [M + H]$^+$ Calcd for $C_{28}H_{33}N_4O_2$ 457.2598; Found 457.2600.

Synthesis of (1H-benzo[d]imidazol-1-yl)(phenyl)methanone (BIPM)

Benzimidazole (500 mg, 0.4 mmol) was dissolved in 20 mL of toluene and Na_2CO_3 (500 mg, 0.5 mmol) and stirred overnight at room temperature. Then, benzoyl chloride (1.8 g, 12 mmol) was dissolved in 10 mL toluene and slowly adding dropwise to the above mixture, and refluxed for 10 h. After cooling to room temperature, the mixture was poured into water and extracted. The organic layer was washed with saturated NaCl solution and dried with anhydrous Na_2SO_4. After filtration and solvent evaporation, the crude residue was purified by silica-gel column chromatography (petroleum ether–ethyl acetate, 15/1~4/1) to give a

white solid, 630 mg, yield 67.0 %. ^1H NMR (400 MHz, $CDCl_3$) δ ^1H NMR (400 MHz, $CDCl_3$) δ 8.24 (s, 1H), 8.21 (dd, *J* = 6.4, 2.6 Hz, 1H), 7.85 (dd, *J* = 6.3, 2.6 Hz, 1H), 7.83 – 7.78 (m, 2H), 7.70 (t, *J* = 7.5 Hz, 1H), 7.60 (t, *J* = 7.6 Hz, 2H), 7.46 (pd, *J* = 7.4, 3.7 Hz, 2H). ^{13}C NMR (100 MHz, $CDCl_3$) δ 1^{13}C NMR (101 MHz, $CDCl_3$) δ 167.2, 144.0, 143.1, 133.3, 132.9, 132.1, 129.6, 129.1, 125.9, 125.4, 120.6, 115.5. HRMS (ESI) m/z: $[M + H]^+$ Calcd for $C_{10}H_9N_2O$ 173.0709; Found 173.0709.

Synthesis of (1H-imidazol-1-yl)(phenyl)methanone (IPM)

Imidazole (500 mg, 0.7 mmol) was dissolved in 20 mL toluene and Na_2CO_3 (930 mg, 0.9 mmol) and stirred overnight at room temperature. Then, benzoyl chloride (3.1 g, 22 mmol) was dissolved in 10 mL toluene and slowly added dropwise to the above mixture, and refluxed for 10 h. After cooling to room temperature, the mixture was poured into water and extracted. The organic layer was washed with saturated NaCl solution and dried with anhydrous Na_2SO_4. After filtration and solvent evaporation, the crude residue was purified by silica-gel column chromatography (petroleum ether–ethyl acetate, 8/1~2/1) to give 890 mg light brown oil, yield 70.4 %. ^1H NMR (400 MHz, $CDCl_3$) δ 8.08 (s, 1H), 7.82 – 7.77 (m, 2H), 7.72 – 7.66 (m, 1H), 7.59 – 7.53 (m, 3H), 7.17 (s, 1H). ^{13}C NMR (100 MHz, $CDCl_3$) δ 166.2, 138.2, 133.7, 131.9, 130.9, 129.8, 129.0, 118.1. HRMS (ESI) m/z: $[M + H]^+$ Calcd for $C_{10}H_8N_3O$ 186.0662; Found 186.0526.

Synthesis of (2-methyl-1H-imidazol-1-yl)(phenyl)methanone (MIPM)

2-methylimidazole (500 mg, 0.6 mmol) was dissolved in 20 mL toluene and Na_2CO_3 (770 mg, 0.7 mmol) and stirred overnight at room temperature. Then, benzoyl chloride (2.6 g, 18 mmol) was dissolved in 10 mL toluene and slowly added dropwise to the above mixture, and refluxed for 10 h. After cooling to room temperature, the mixture was poured into water and extracted. The organic layer was washed with saturated NaCl solution and dried with anhydrous Na_2SO_4. After filtration and solvent evaporation, the crude residue was purified by silica-gel column chromatography (petroleum ether–ethyl acetate, 6/1~2/1) to give 810 mg colorless oil, yield 71.4 %. ^1H NMR (400 MHz, $CDCl_3$) δ 7.76 – 7.69 (m, 2H), 7.60 (t, *J*

= 7.5 Hz, 1H), 7.48 (t, *J* = 7.7 Hz, 2H), 7.02 (d, *J* = 1.7 Hz, 1H), 6.84 (d, *J* = 1.7 Hz, 1H), 2.64 (s, 3H). ^{13}C NMR (100 MHz, $CDCl_3$) δ 167.9, 148.5, 133.4, 132.8, 129.8, 128.7, 127.6, 120.0, 16.6. HRMS (ESI) m/z: $[M + H]^+$ Calcd for $C_{11}H_{11}N_2O$ 187.0866; Found 187.0865.

Synthesis of (3-methyl-1H-1,2,4-triazol-1-yl)(phenyl)methanone (MTPM)

3-methyl-1H-1,2,4-triazole (500 mg, 0.6 mmol) was dissolved in 20 mL toluene and Na_2CO_3 (770 mg, 0.7 mmol) and stirred overnight with room temperature. Then, benzoyl chloride (2.5 g, 18 mmol) was dissolved in 10 mL of toluene and slowly added dropwise to the above mixture, and refluxed for 10 h. After cooling to room temperature, the mixture was poured into water and extracted. The organic layer was washed with saturated NaCl solution and dried with anhydrous Na_2SO_4. After filtration and solvent evaporation, the crude residue was purified by silica-gel column chromatography (petroleum ether–ethyl acetate, 20/1~5/1) to give 710 mg colorless oil, yield 63.0 %. ^{1}H NMR (400 MHz, $CDCl_3$) δ 8.15 (dd, *J* = 8.4, 1.2 Hz, 2H), 7.66 – 7.58 (m, 1H), 7.48 (t, *J* = 7.8 Hz, 3H), 2.45 (s, 3H). ^{13}C NMR (100 MHz, $CDCl_3$) δ 1 164.4, 163.4, 150.8, 146.2, 134.1, 131.6, 128.5, 15.6. HRMS (ESI) m/z: $[M - H]^-$ Calcd for Calcd for $C_{10}H_8N_3O$ 186.0662; Found 186.0526.

9. The ^{1}H NMR, ^{13}C NMR and HRMS spectra of ASX, BIPM, IPM, MIPM and MTPM

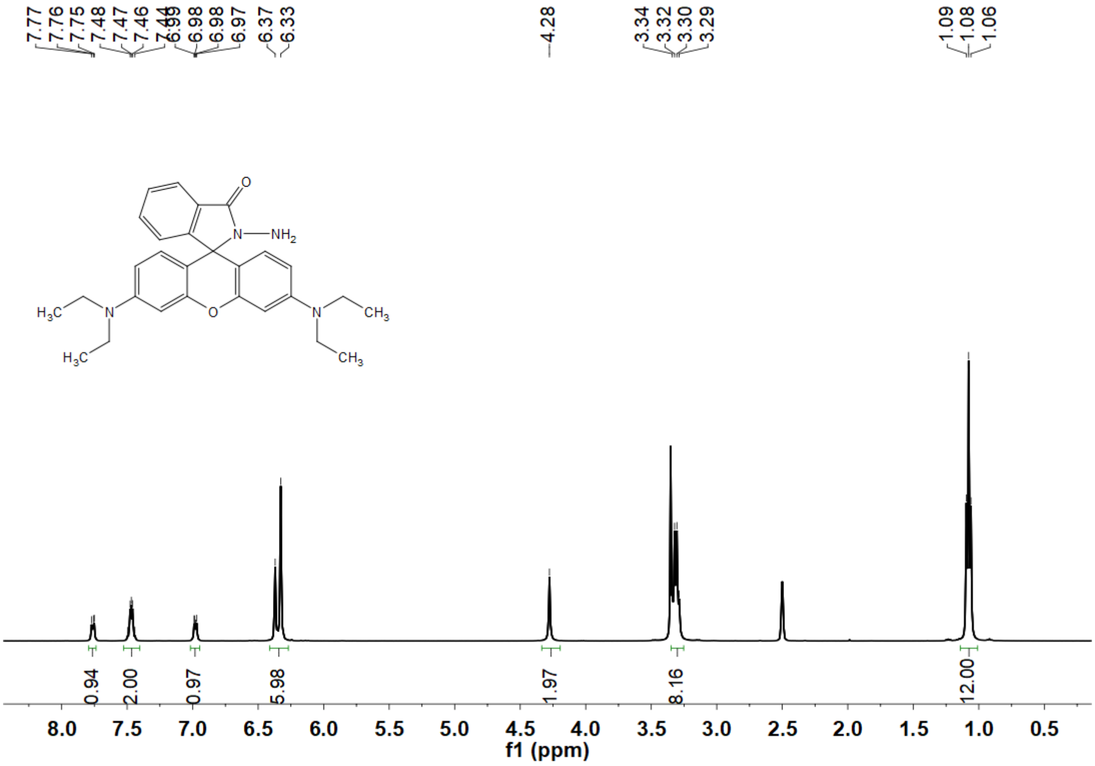

Supplementary Fig. 25 ^{1}H NMR of ASX in DMSO-d_6 (400 MHz).

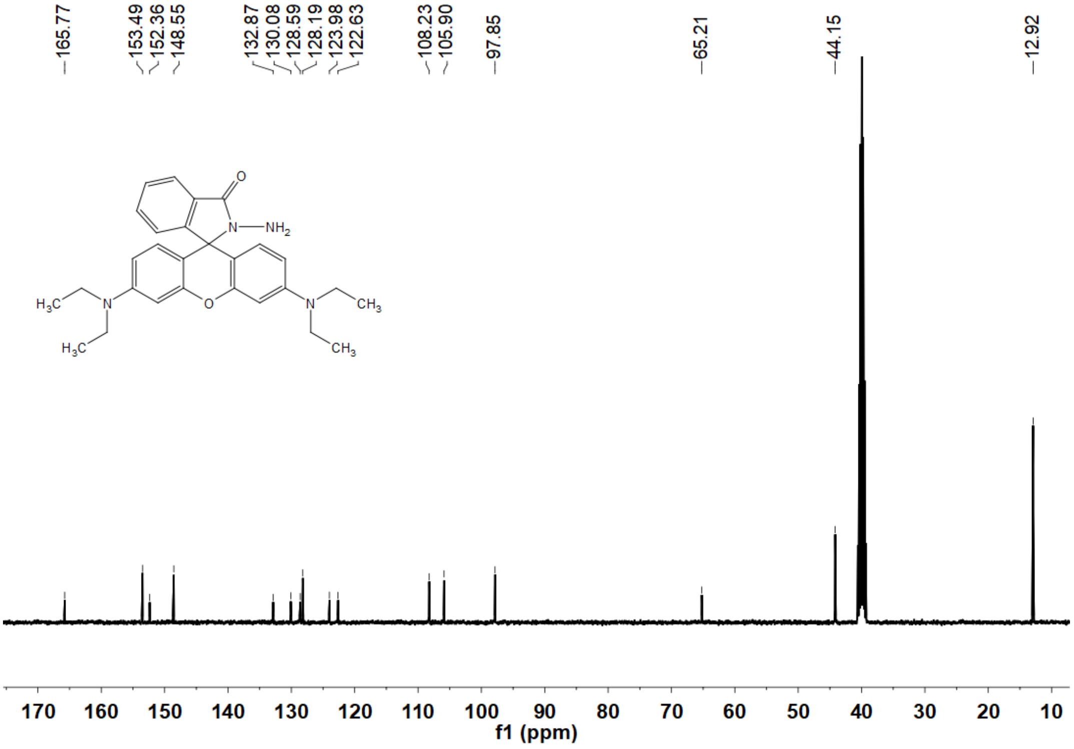

Supplementary Fig. 26 ^{13}C NMR of ASX in DMSO-d_6 (100 MHz).

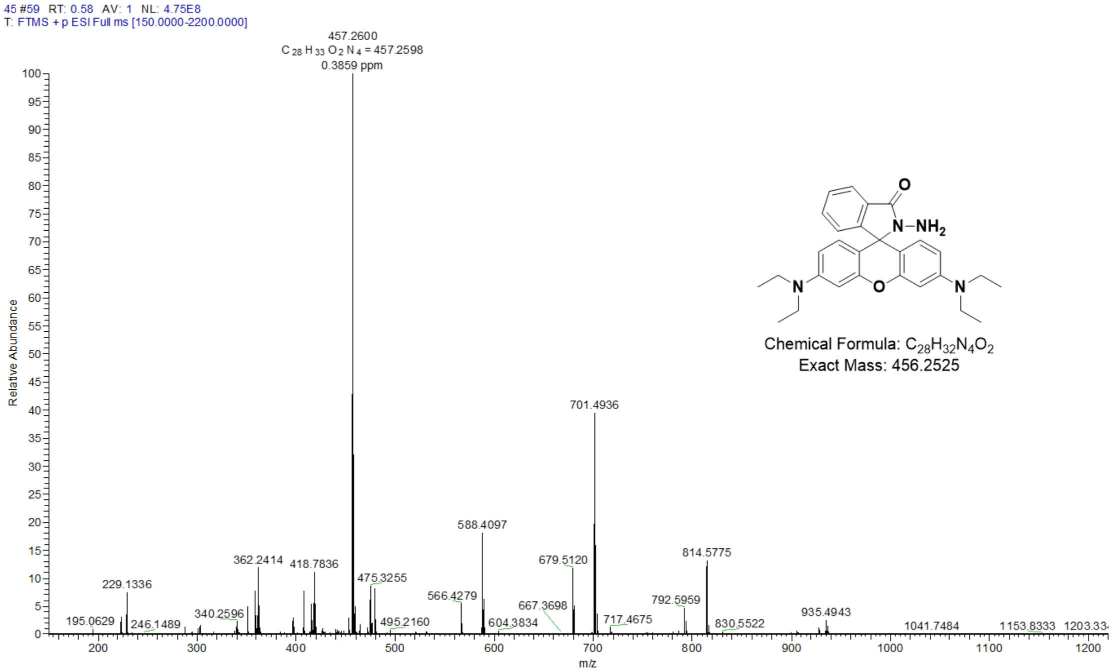

Supplementary Fig. 27 HRMS spectrum of ASX.

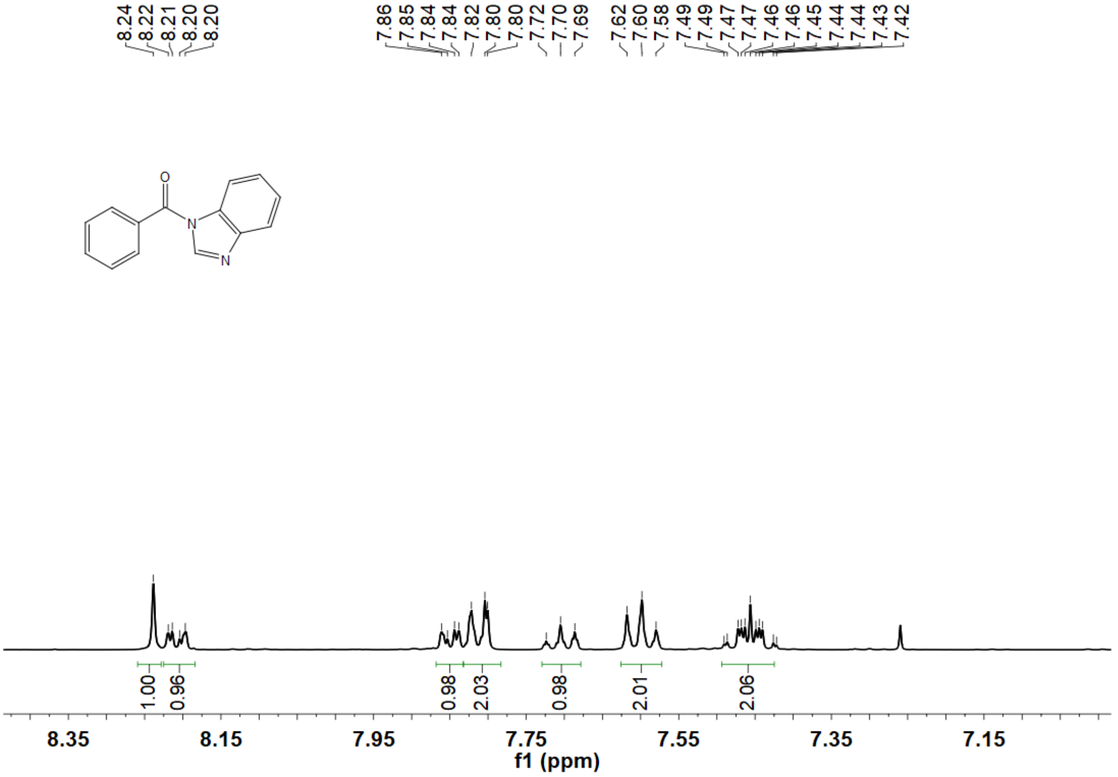

Supplementary Fig. 28 ^{1}H NMR of BIPM in $CDCl_3$ (400 MHz).

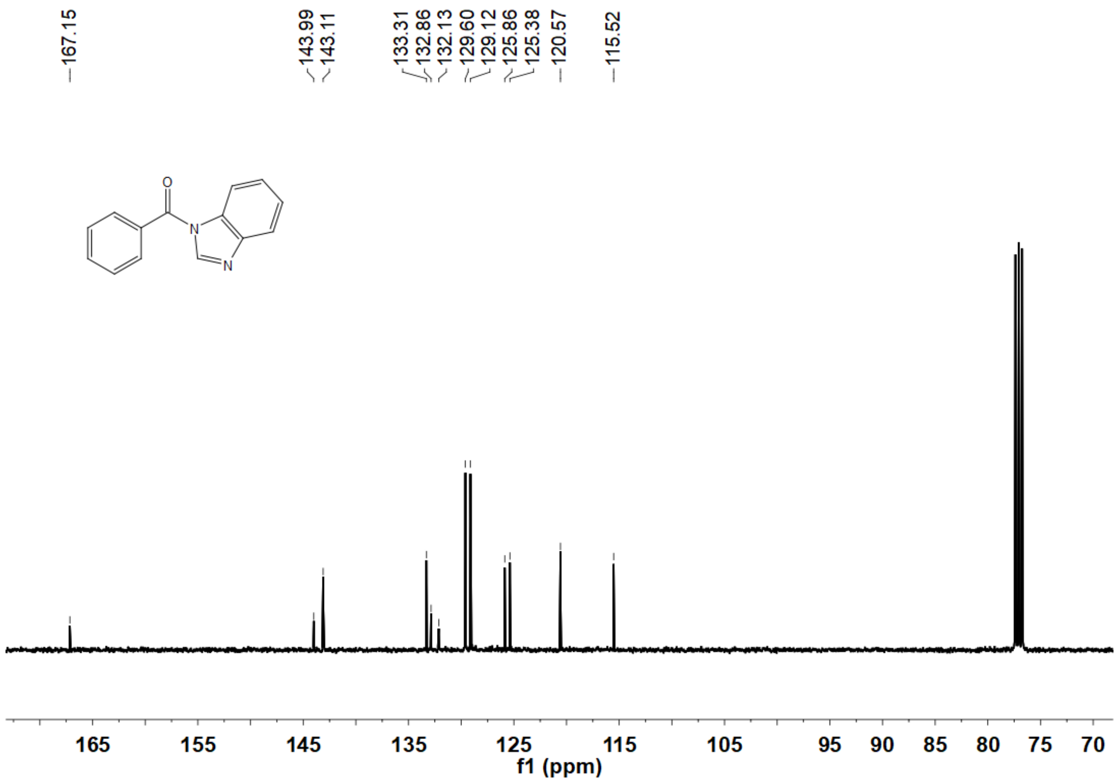

Supplementary Fig. 29 ^{13}C NMR of BIPM in $CDCl_3$ (100 MHz).

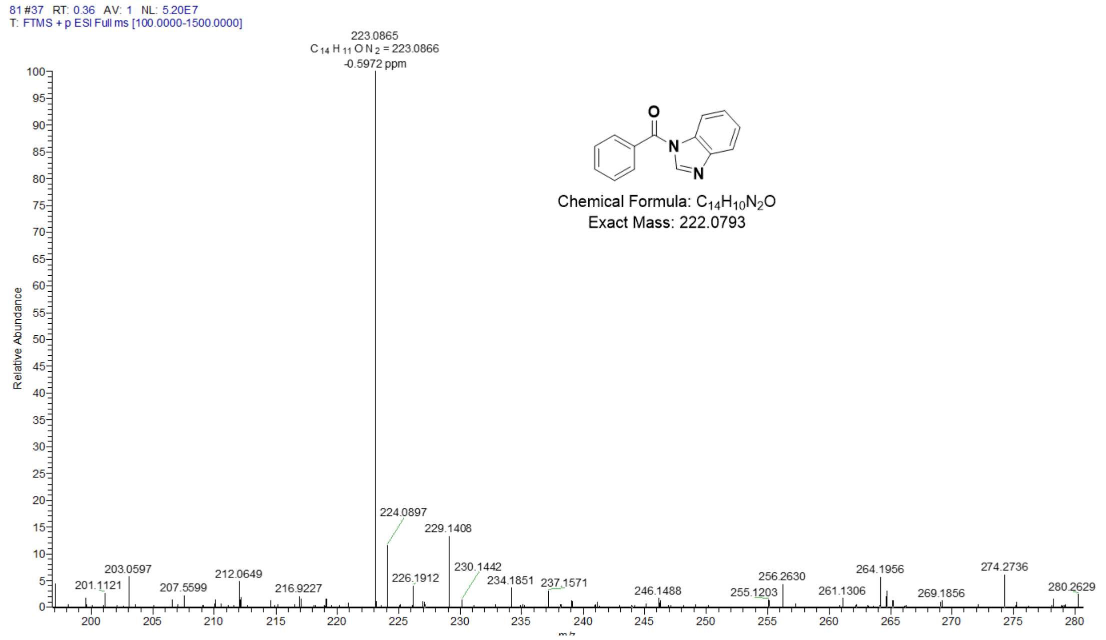

Supplementary Fig. 30 HRMS spectrum of BIPM.

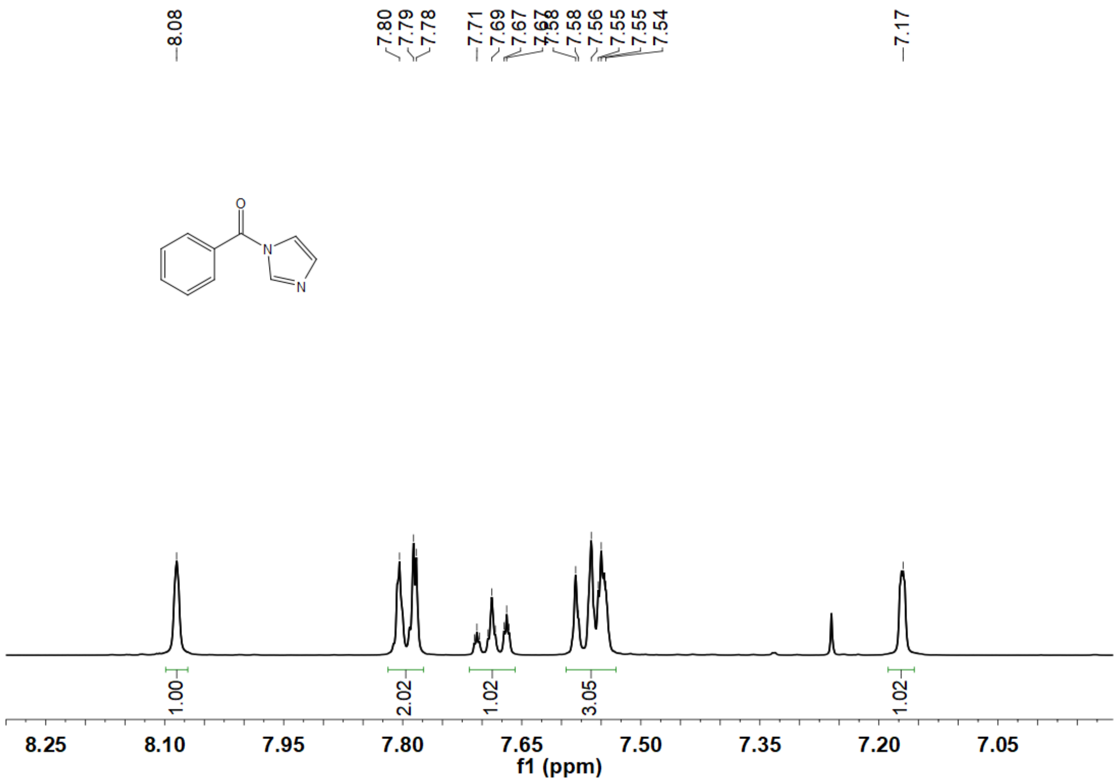

Supplementary Fig. 31 ^{1}H NMR of IPM in $CDCl_3$ (400 MHz).

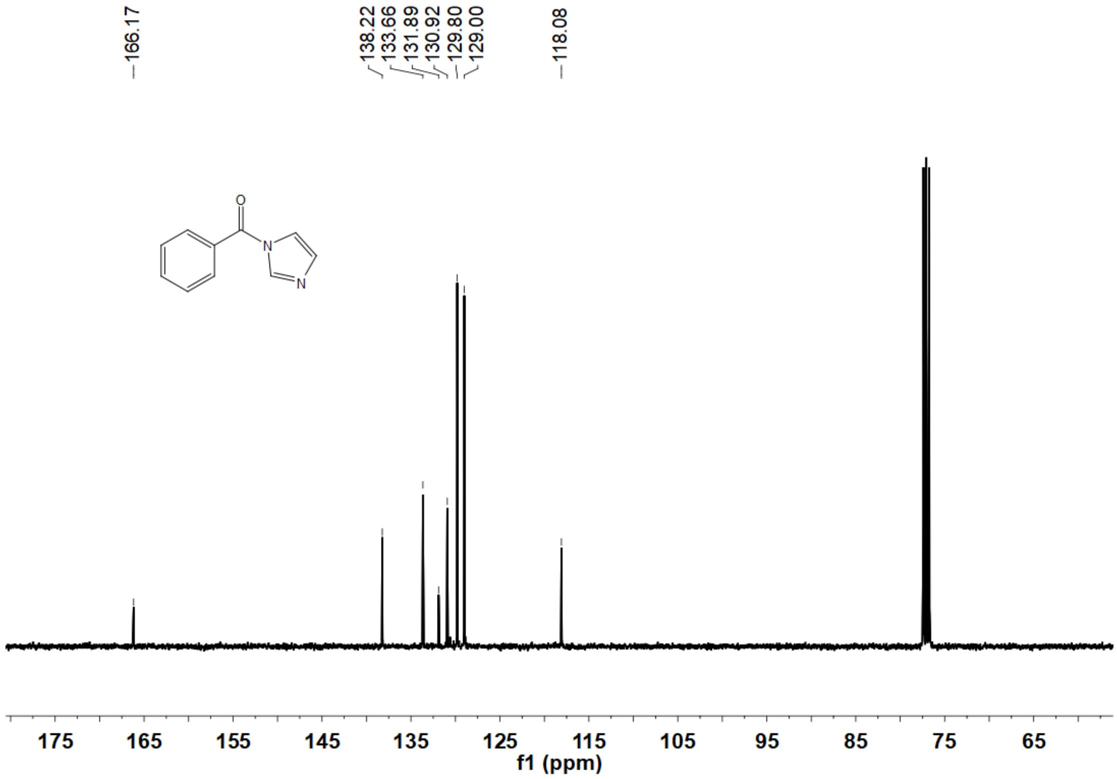

Supplementary Fig. 32 ^{13}C NMR of IPM in $CDCl_3$ (100 MHz).

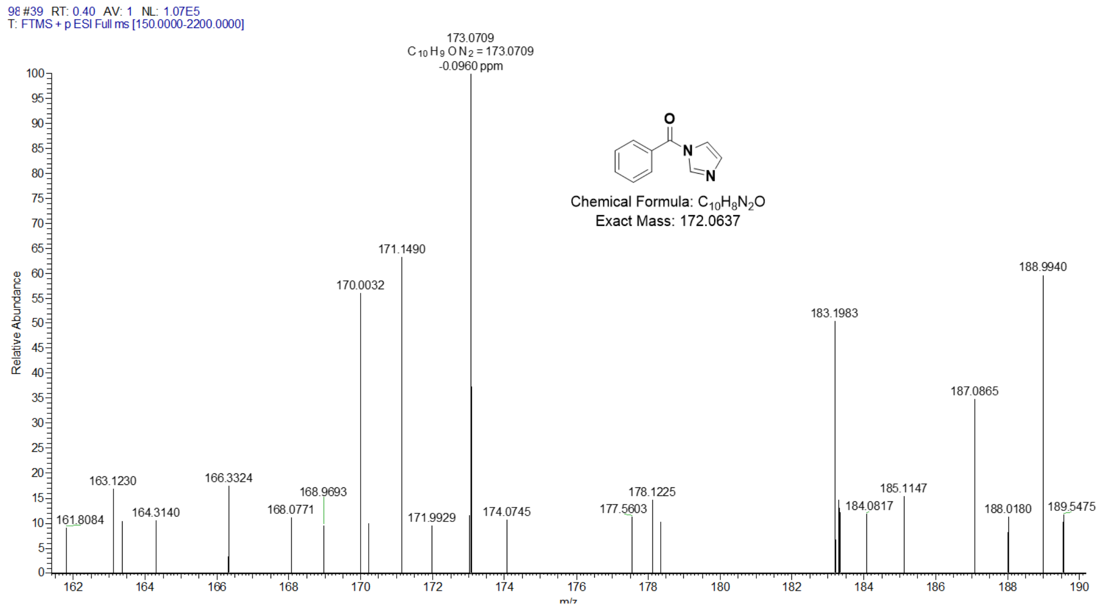

Supplementary Fig. 33 HRMS spectrum of IPM.

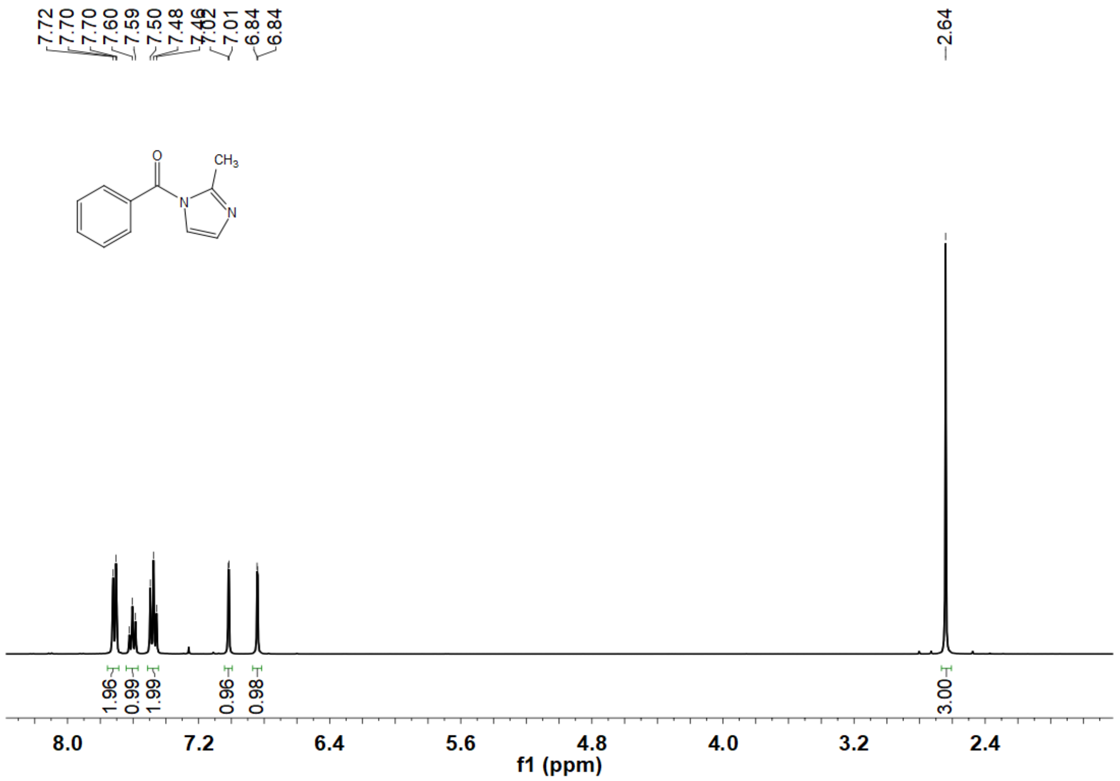

Supplementary Fig. 34 ^{1}H NMR of MIPM in $CDCl_3$ (400 MHz).

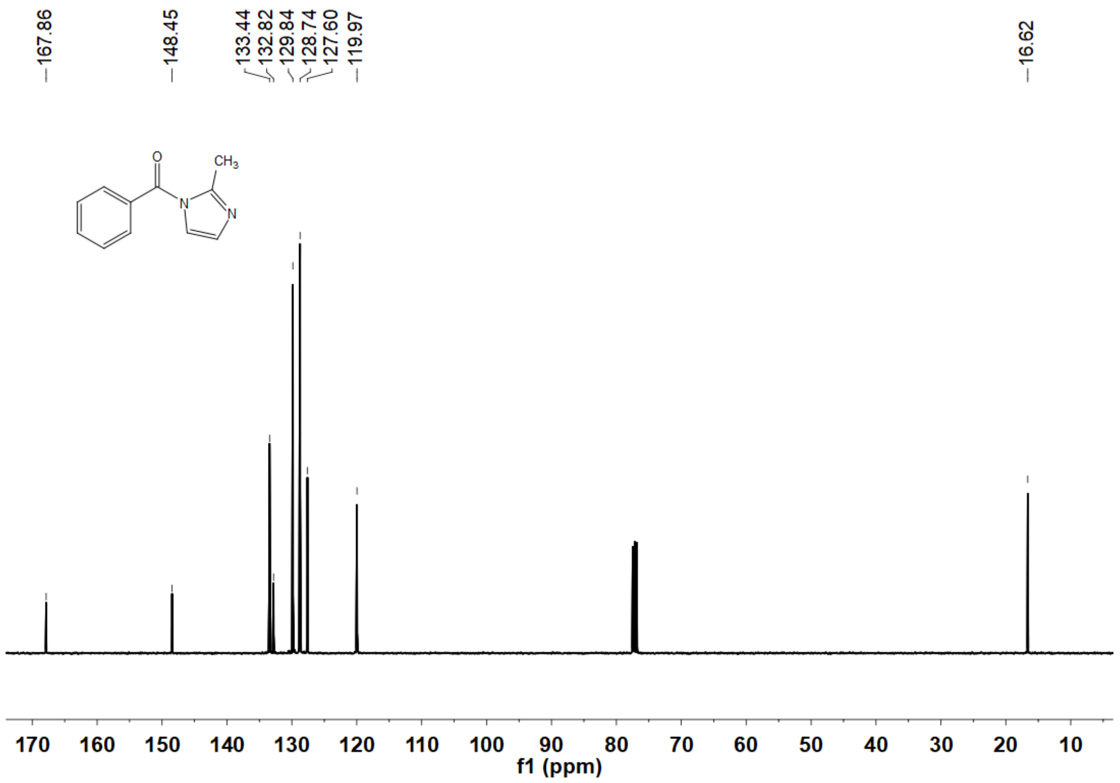

Supplementary Fig. 35 ^{13}C NMR of MIPM in $CDCl_3$ (100 MHz).

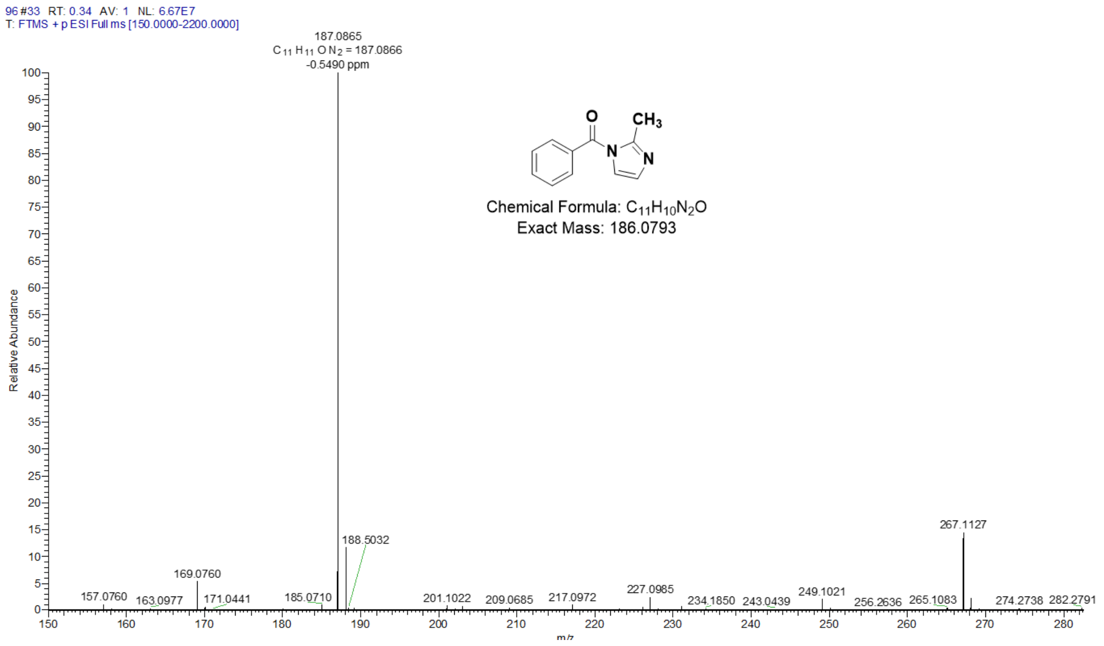

Supplementary Fig. 36 HRMS spectrum of MIPM.

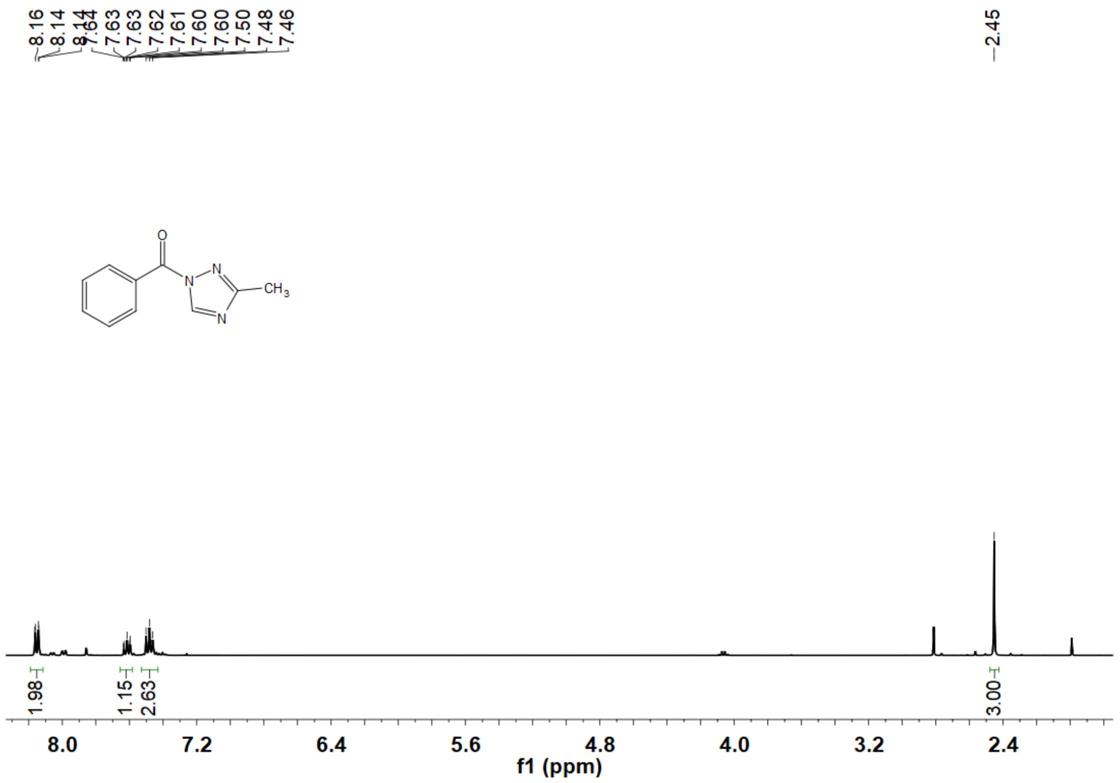

Supplementary Fig. 37 ^{1}H NMR of MTPM in $CDCl_3$ (400 MHz).

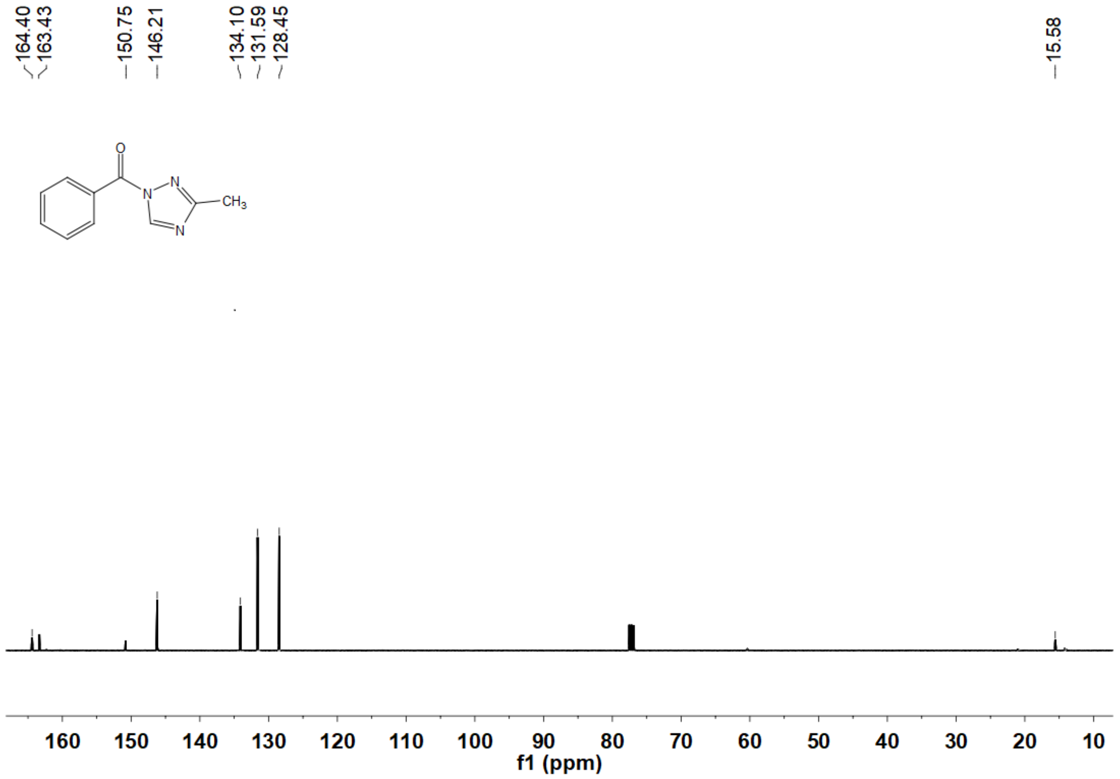

Supplementary Fig. 38 ^{13}C NMR of MTPM in $CDCl_3$ (100 MHz).

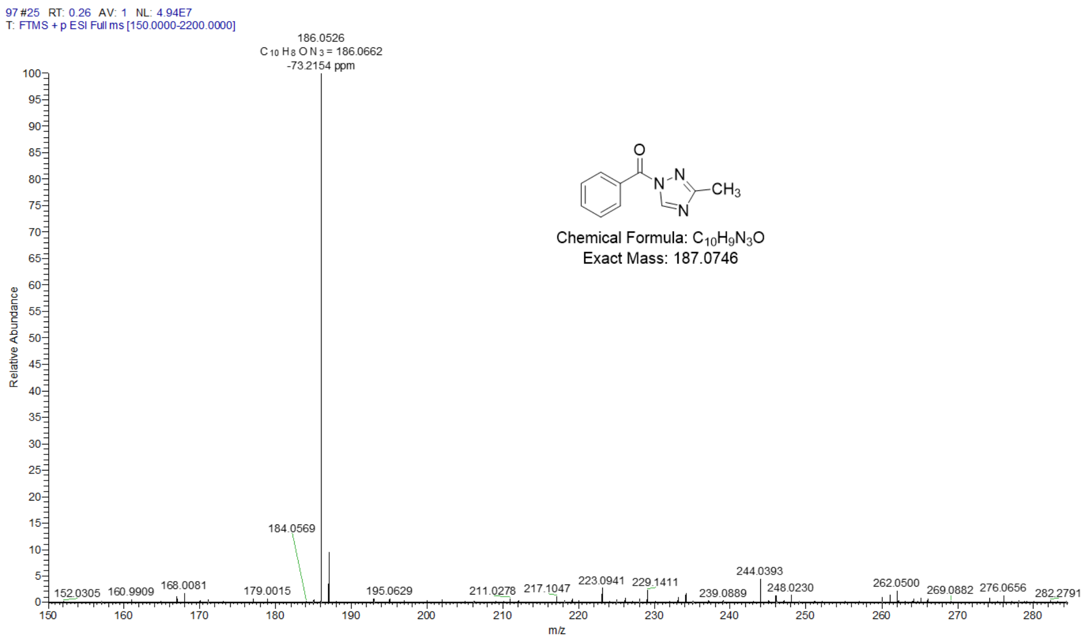

Supplementary Fig. 39 HRMS spectrum of MTPM.

10. PL spectra of ASX, BIPM, IPM, MIPM and MTPM

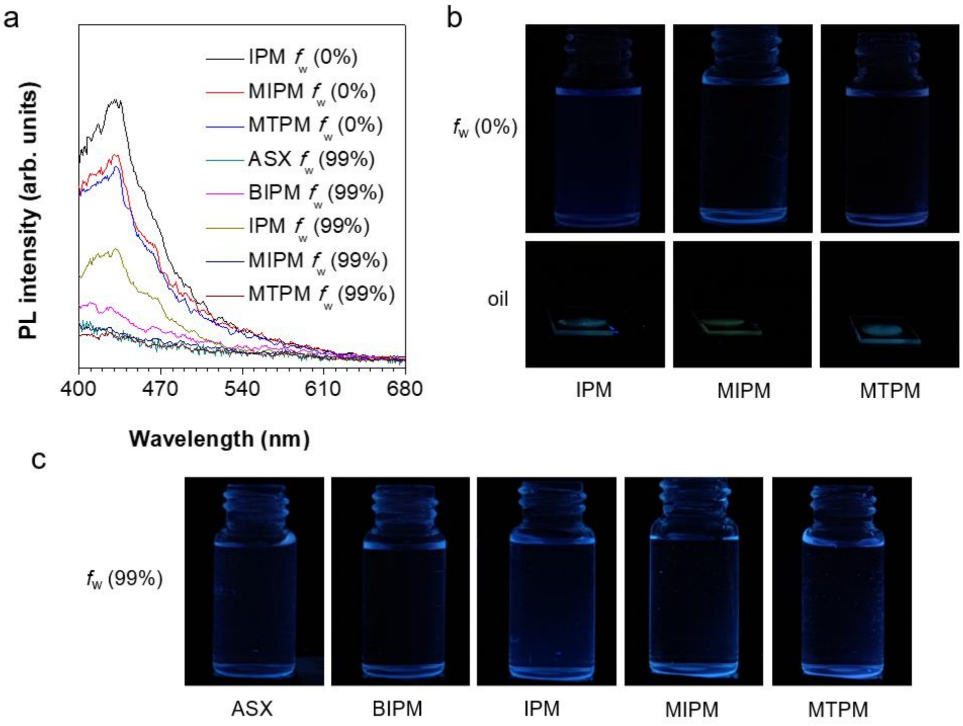

Supplementary Fig. 40 (a) PL spectra of IPM, MIPM and MTPM in THF, and ASX, BIPM, IPM, MIPM and MTPM in THF/water mixtures with f_w = 99%. Concentration (c) = 1×10^{-5} M, excitation wavelength (λ_{ex}) = 365 nm. (b) Fluorescence pictures of IPM, MIPM and MTPM in THF and oil. (c) Fluorescence pictures of ASX, BIPM, IPM, MIPM and MTPM in THF/water mixtures with f_w = 99%.

11. Excitation spectra of BISX, ISX, MISX and MTSX

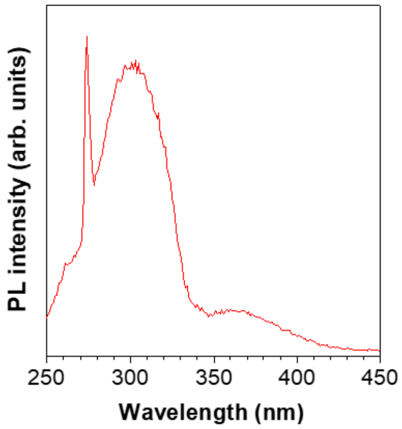

Supplementary Fig. 41 Excitation spectrum of BISX in THF. Concentration (c) = 1×10^{-5} M, emission wavelength (λ_{ex}) = 540 nm.

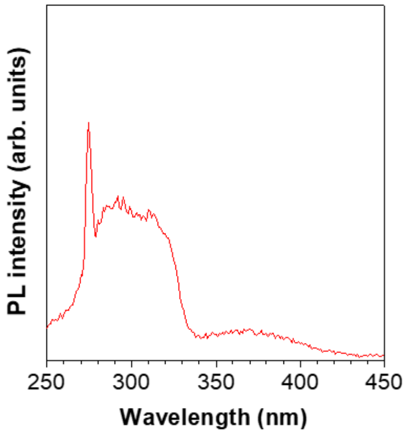

Supplementary Fig. 42 Excitation spectrum of ISX in THF. Concentration (c) = 1×10^{-5} M, emission wavelength (λ_{ex}) = 540 nm.

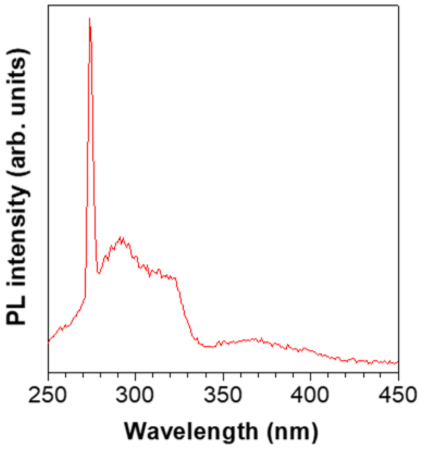

Supplementary Fig. 43 Excitation spectrum of MISX in THF. Concentration (c) = 1×10^{-5} M, emission wavelength (λ_{ex}) = 540 nm.

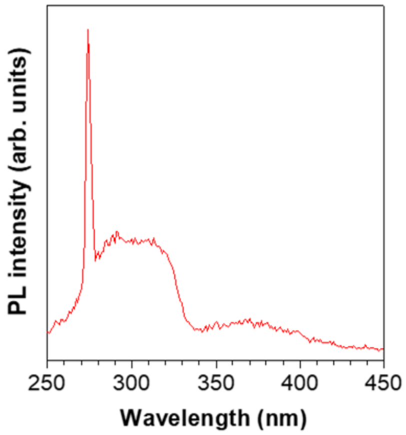

Supplementary Fig. 44 Excitation spectrum of MTSX in THF. Concentration (c) = 1×10^{-5} M, emission wavelength (λ_{ex}) = 540 nm.

12. Intermolecular interactions of BISX, ISX, MISX and MTSX crystals

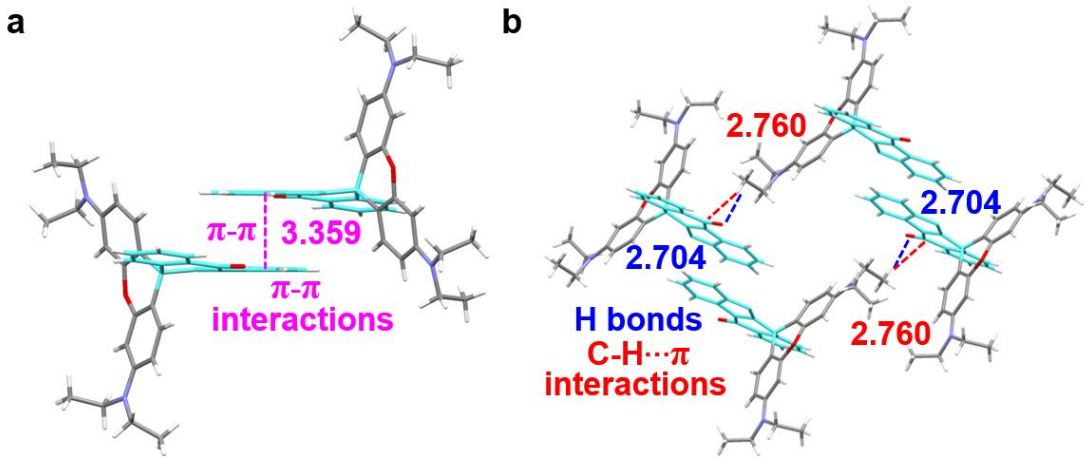

Supplementary Fig. 45 (a) The π-π intermolecular interactions (purple square dotted lines) of BISX. (b) The C-H···π intermolecular interactions (red square dotted lines) and H-bonds (blue square dotted lines) of BISX.

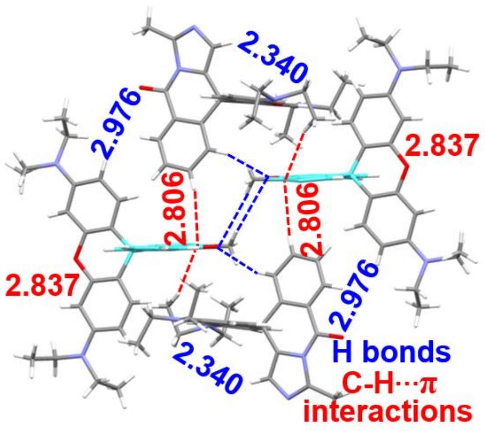

Supplementary Fig. 46 The intermolecular interactions (C-H···π red square dotted lines) and H-bonds

(blue square dotted lines) of ISX.

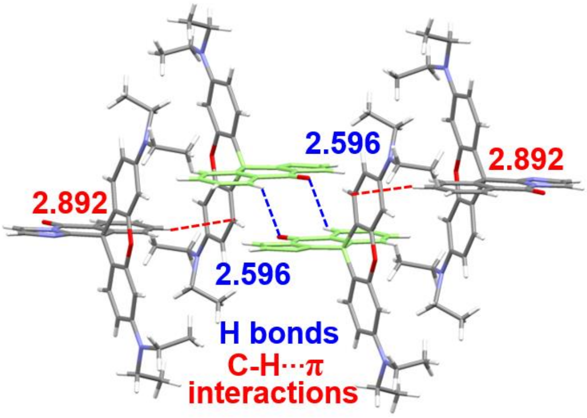

Supplementary Fig. 47 The intermolecular interactions (C-H···π red square dotted lines) and H-bonds (blue square dotted lines) of MISX.

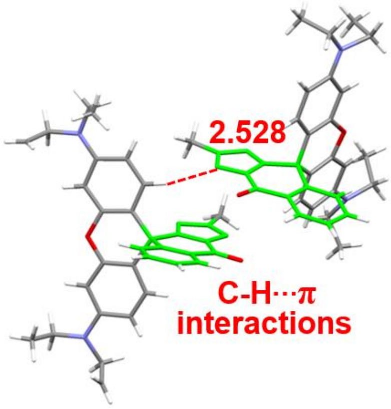

Supplementary Fig. 48 The intermolecular interactions (C-H···π red square dotted lines) of MTSX.

13. The UV-vis absorbance spectra in the film of BISX, ISX, MISX and MTSX in PMMA

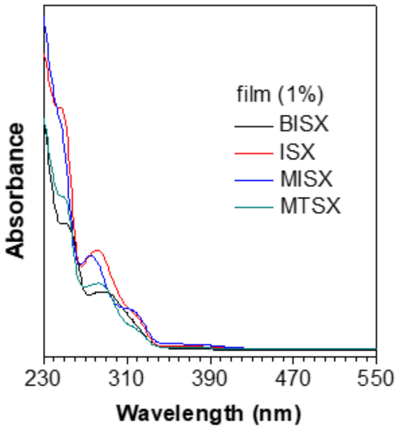

Supplementary Fig. 49 UV-vis absorption spectra of BISX, ISX, MISX and MTSX doped in PMMA film with 1% weight.

14. Theoretical calculation

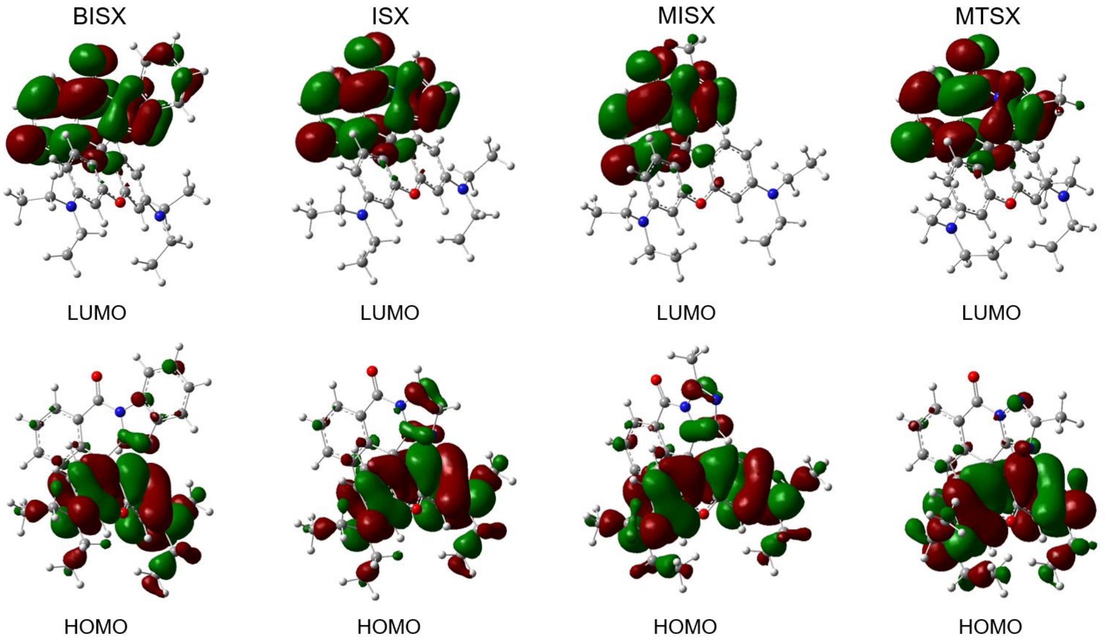

Supplementary Fig. 50 The ground state distribution of frontier molecular orbitals of BISX, ISX, MISX and MTSX in THF solutions.

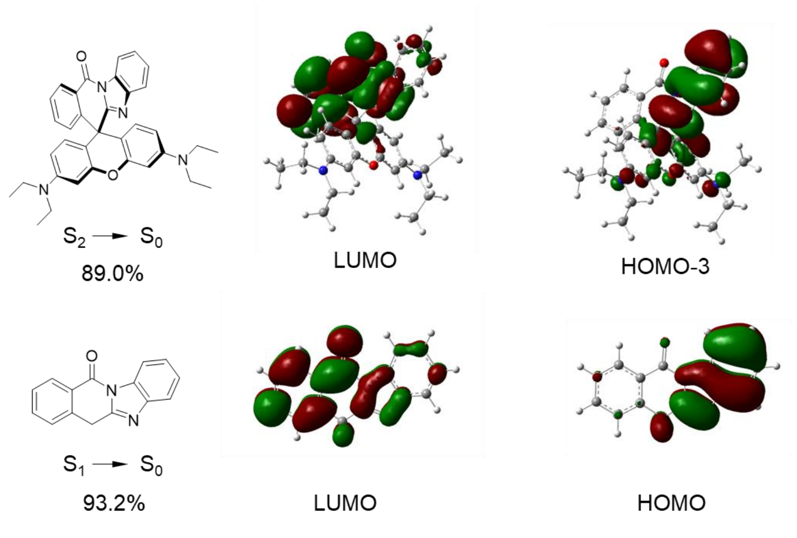

Supplementary Fig. 51 The ground state distribution of frontier molecular orbitals of BISX (top), and isoquinolinone (bottom) molecules in THF solutions.

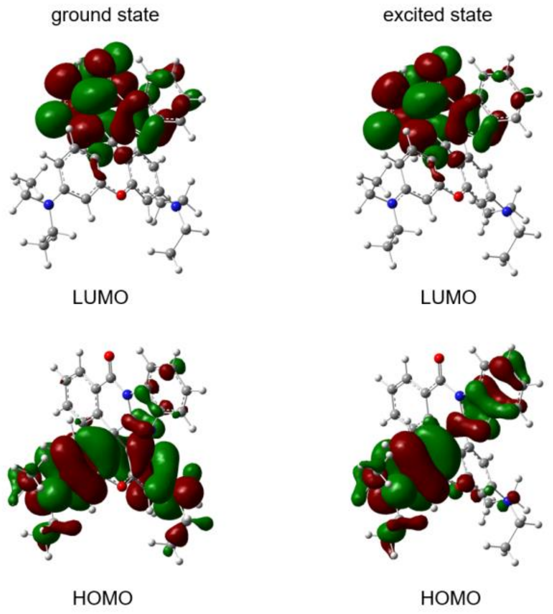

Supplementary Fig. 52 Frontier molecular orbitals of BISX in the crystal state.

Supplementary Tab. 3 The oscillator strengths (f) of the S1 state of BISX and isoquinolinone compound

	Oscillator strength (*f*)
$BISX_{THF\ solution,\ abs}$	0.0115
$BISX_{THF\ solution,\ CTD\text{-}A}$	0.0042
$isoquinolinone_{THF\ solution,\ CTA}$	0.0159

Supplementary Tab. 4 Theoretical calculations for fluorescence behaviors of BISX in the different states.

	$\lambda_{computational}$ (nm)	Energy $gap_{S0\text{-}S1,\ computational}$ (eV)
$BISX_{THF\ solution,\ abs}$	309.55	4.01
$BISX_{THF\ solution,\ CTD\text{-}A}$	397.79	3.12
$BISX_{aggregates,\ abs}$	310.21	4.00
$BISX_{aggregates,\ CTD\text{-}A}$	393.24	3.15

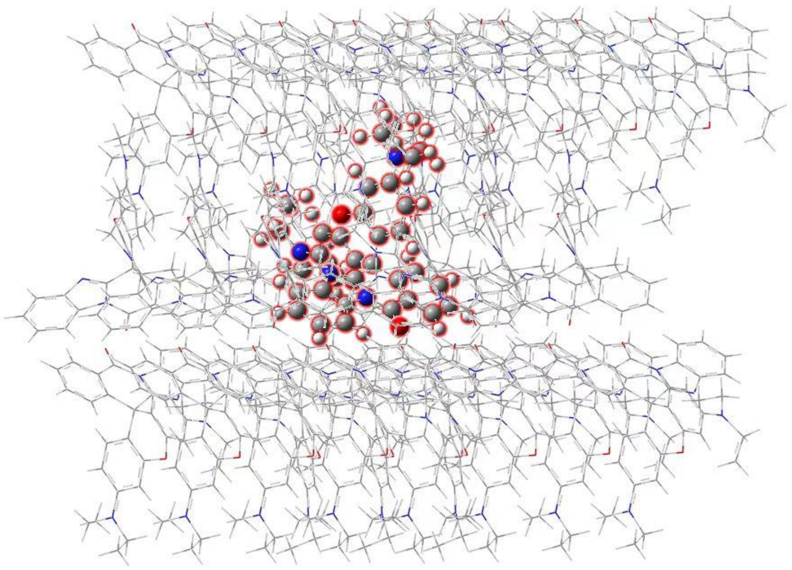

Supplementary Fig. 53 ONIOM model of BISX. The central monomer is QM part for the high layer and the others are MM part for the low layer.

15. BISX treated with TFA and TEA

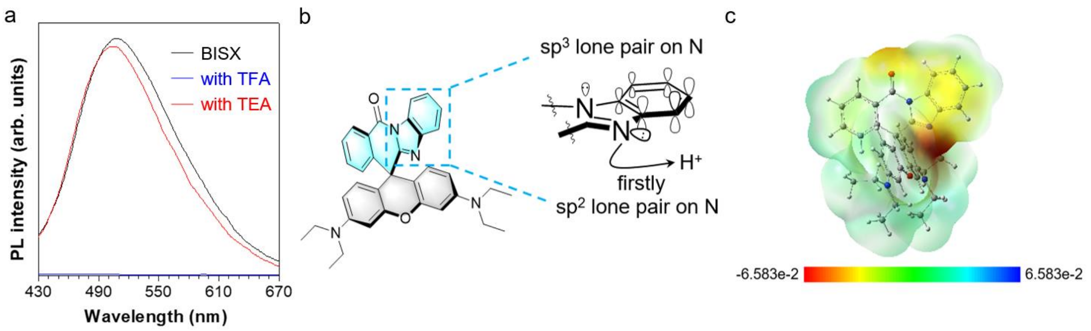

Supplementary Fig. 54 (a) PL spectra of BISX treated with TFA and TEA subsequently. (b) The possible mechanism of BISX treated with TFA. (c) Calculated electron density mapped with electrostatic potential of BISX based on optimized structure. The negative part is shown in red and positive part is shown in blue.

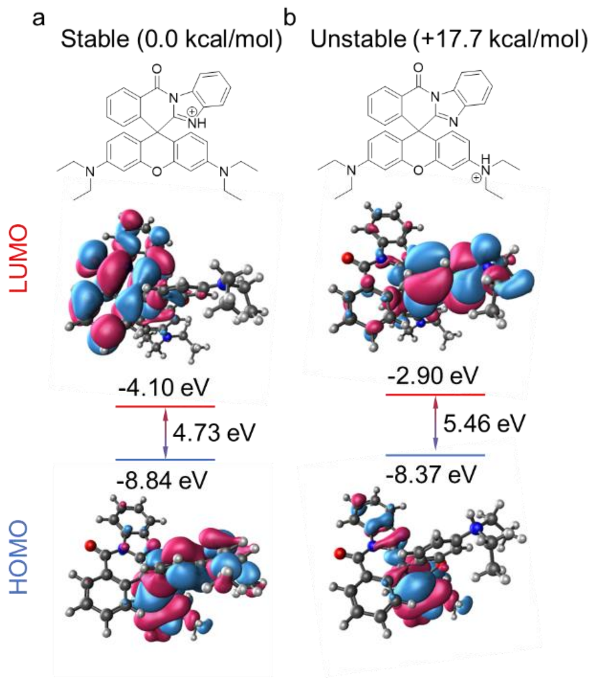

Supplementary Fig. 55 Frontier molecular orbitals of protonated compounds of BISX based on their optimized ground-state geometries for protonated sp2 hybridized nitrogen (a) and the protonated nitrogen from diethylamine substituents (b).

16. Application in the detection of amines released by clams, crayfish and fish spoilage

BISX (2 mg) was dissolved in 1 mL THF and wetted filter paper. Then, let the filter paper dry, it was fumed by trifluoroacetic acid (TFA). Fresh clams, crayfish and fish were collected from seafood market and respectively TFA-treated filter paper was put into in black plastic-wrapped container and kept at room temperature.

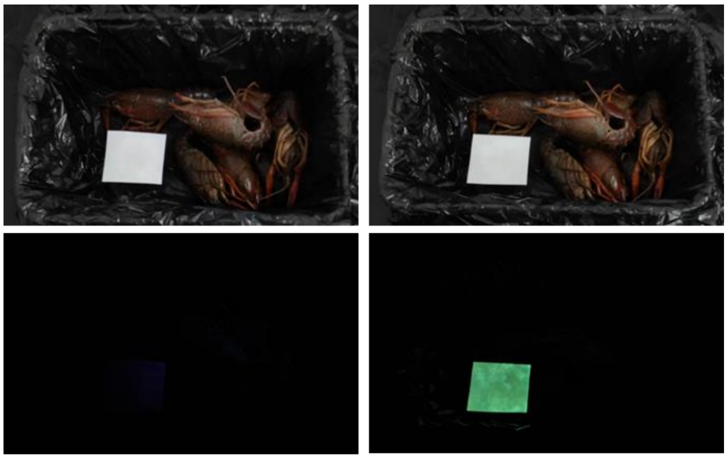

0 h 12 h

Supplementary Fig. 56 Spoilage detection of crayfish in sealed packages for 12 h at room temperature using BISX. Photographs taken under daylight (upper) and 365 nm UV irradiation (bottom).

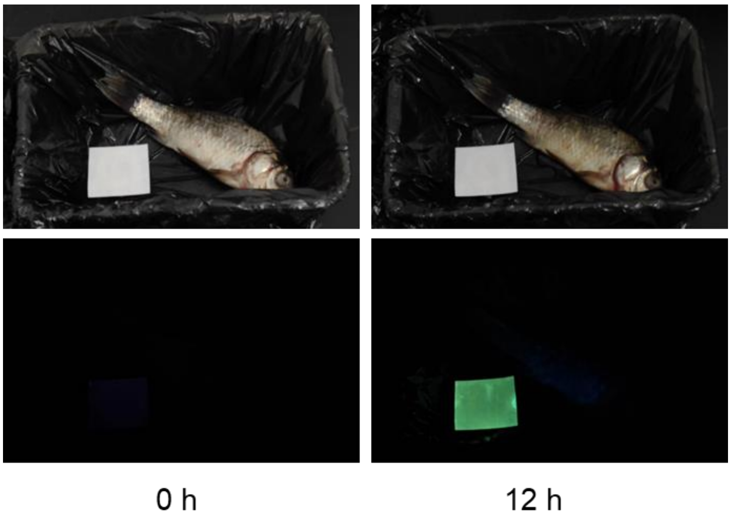

Supplementary Fig. 57 Spoilage detection of fish in sealed packages for 12 h at room temperature using BISX. Photographs taken under daylight (upper) and 365 nm UV irradiation (bottom).
